# Hematopoietic Stem Cell Transplantation in Primary Immunodeficiency Diseases: Current Status and Future Perspectives

**DOI:** 10.3389/fped.2019.00295

**Published:** 2019-08-08

**Authors:** Riccardo Castagnoli, Ottavia Maria Delmonte, Enrica Calzoni, Luigi Daniele Notarangelo

**Affiliations:** ^1^Laboratory of Clinical Immunology and Microbiology, Division of Intramural Research, National Institute of Allergy and Infectious Diseases, National Institutes of Health, Bethesda, MD, United States; ^2^Department of Pediatrics, Foundation IRCCS Policlinico San Matteo, University of Pavia, Pavia, Italy; ^3^Department of Molecular and Translational Medicine, A. Nocivelli Institute for Molecular Medicine, University of Brescia, Brescia, Italy

**Keywords:** primary immunodeficiency diseases (PID), hematopoietic stem cell transplantation, transplantation outcomes, immune dysregulation, severe combined immunodeficiency, graft manipulation, conditioning regimens, precision medicine

## Abstract

Primary immunodeficiencies (PID) are disorders that for the most part result from mutations in genes involved in immune host defense and immunoregulation. These conditions are characterized by various combinations of recurrent infections, autoimmunity, lymphoproliferation, inflammatory manifestations, atopy, and malignancy. Most PID are due to genetic defects that are intrinsic to hematopoietic cells. Therefore, replacement of mutant cells by healthy donor hematopoietic stem cells (HSC) represents a rational therapeutic approach. Full or partial ablation of the recipient's marrow with chemotherapy is often used to allow stable engraftment of donor-derived HSCs, and serotherapy may be added to the conditioning regimen to reduce the risks of graft rejection and graft versus host disease (GVHD). Initially, hematopoietic stem cell transplantation (HSCT) was attempted in patients with severe combined immunodeficiency (SCID) as the only available curative treatment. It was a challenging procedure, associated with elevated rates of morbidity and mortality. Overtime, outcome of HSCT for PID has significantly improved due to availability of high-resolution HLA typing, increased use of alternative donors and new stem cell sources, development of less toxic, reduced-intensity conditioning (RIC) regimens, and cellular engineering techniques for graft manipulation. Early identification of infants affected by SCID, prior to infectious complication, through newborn screening (NBS) programs and prompt genetic diagnosis with Next Generation Sequencing (NGS) techniques, have also ameliorated the outcome of HSCT. In addition, HSCT has been applied to treat a broader range of PID, including disorders of immune dysregulation. Yet, the broad spectrum of clinical and immunological phenotypes associated with PID makes it difficult to define a universal transplant regimen. As such, integration of knowledge between immunologists and transplant specialists is necessary for the development of innovative transplant protocols and to monitor their results during follow-up. Despite the improved outcome observed after HSCT, patients with severe forms of PID still face significant challenges of short and long-term transplant-related complications. To address this issue, novel HSCT strategies are being implemented aiming to improve both survival and long-term quality of life. This article will discuss the current status and latest developments in HSCT for PID, and present data regarding approach and outcome of HSCT in recently described PID, including disorders associated with immune dysregulation.

## Overview on Hematopoietic Stem Cell Transplantation in Primary Immunodeficiency Diseases

Primary immunodeficiencies (PID) are for the most part monogenic disorders resulting from mutations in genes involved in immune host defense and immunoregulation. These conditions are characterized by various combinations of recurrent infections, autoimmunity, lymphoproliferation, inflammatory manifestations, atopy, and malignancy. By now, more than 300 genetic causes of PID have been identified. The International Union of Immunological Societies (IUIS) Inborn Errors of Immunity Committee have classified them in 9 groups according to their clinical and immunological phenotype [Table 1; (1, 2)]. Most PID are due to genetic defects that are intrinsic to hematopoietic cells. Therefore, replacement of mutant cells by healthy donor hematopoietic stem cells (HSCs) represents a rational therapeutic approach. In particular, patients with severe combined immunodeficiency (SCID) represent a medical emergency, as these infants are highly susceptible to life-threatening infections. In these cases, allogenic hematopoietic stem cell transplantation (HSCT) provides a life-saving and curative treatment. Soon after the report of the human major histocompatibility complex (MHC) in 1967 (3), the first successful HSCT in PID included SCID and Wiskott-Aldrich Syndrome (WAS) (4, 5). Overtime outcome of HSCT for PID has significantly ameliorated and survival for conventional PID is now reaching 90% (6, 7).

**Table 1:** Classification of PID according to IUIS Primary Immunodeficiency Diseases Committee Report on Inborn Errors of Immunity (1, 2).

**I. Immunodeficiencies affecting cellular and humoral immunity**			
a. Severe Combined Immunodeficiency (SCID) defined by CD3 T cell lymphopenia			
T– B+ NK–	T– B+ NK+	T– B– NK-	T– B– NK+
*IL2RG* (SCID-XL)	*IL7R*	*ADA*	*LIG4*
*JAK3*	*CD3δ*	*AK2* (Reticular dysgenesis)	*RAG1*
	*CD3ε*		*RAG2*
	*CD3ζ*		*DCLRE1C* (Artemis deficiency)
	*CORO1A*		*NHEJ1* (Cernunnos XLF)
	*PTPRC* (CD45 deficiency)		*PRKDC* (DNA-PKcs deficiency)
	*FOXN1*		
b. Combined Immunodeficiencies (CID) generally less profound than SCID			
**II. CID with associated or syndromic features**			
**III. Predominantly antibody deficiencies**			
a. Hypogammaglobulinemia			
b. Other antibody deficiencies			
**IV. Diseases of immune dysregulation**			
a. Hemophagocytic Lymphohistiocytosis (HLH)			
b. EBV susceptibility			
c. Syndromes with autoimmunity			
d. Immune dysregulation with colitis			
**V. Congenital defects of phagocyte number, function or both**			
a. Neutropenia			
b. Functional defects			
**VI. Defects in intrinsic and innate immunity**			
a. Predisposition to invasive bacterial infections (pyogenes)			
b. Predisposition to parasitic and fungal infections			
c. Mendelian susceptibility to mycobaterial disease (MSMD)			
d. Predominant susceptibility to viral infection			
**VII. Auto-inflammatory disorders**			
**VIII. Complement deficiencies**			
**IX. Phenocopies of PID**			

*IL2RG, interleukin 2 receptor subunit gamma; JAK3, Janus kinase 3; IL7R, interleukin 7 receptor; CD3δ, CD3δ molecule; CD3ε, CD3ε molecule; CD3ς, CD3ς molecule; CORO1A, coronin 1A; PTPRC, protein tyrosine phosphatase, receptor type C; FOXN1, Forkhead box N1; ADA, adenosine deaminase; AK2, adenylate kinase 2; LIG4, DNA ligase 4; RAG1, recombination activating 1; RAG2, recombination activating 2; DCLRE1C, DNA cross-link repair 1C; NHEJ1, non-homologous end joining factor 1; PRKDC, protein kinase, DNA-activated, catalytic subunit*.

The progresses in the **understanding of the pathological bases of PID and the general principles of HSCT** have been crucial for outcome improvement. In particular, PID stemming from defects in the hematopoietic compartment can be corrected by HSCT. However, immunological diseases due to thymic stromal or other extra-hematopoietic defects are unlikely to be cured by HSCT. If a thymic stromal defect is present, the hematopoietic stem cells cannot undergo a functional maturation in the thymus, thus resulting in poor immune reconstitution and poor HSCT outcomes. Table 2 summarizes classical indications for HSCT in PID. However, it is now clear that decisions regarding the indication and time to transplant must carefully consider the risks of HSCT against the risks of further disease evolution and must be individualized not only on the basis of the specific PID but also on the characteristics of the single patient. For example, PID adult survivors that are currently treated with conservative treatment but whose clinical condition are deteriorating represent a group of patients where transplant decisions are often challenging. Nonetheless, Fox et al. reported on 29 adults with various PID who underwent HSCT with an overall survival of 85% (8), indicating that HSCT should be taken into account also for this group of patients.

**Table 2:** Indications for HSCT in PID.

**HSCT curative**	**HSCT partially curative**	**HSCT controversial**
SCID	Cartilage Hair Hypoplasia	CVID
CID^∧^	PGM3 deficiency	Agammaglobulinemia
CGD	STAT1-GOF	Complement deficiencies (other than C1q deficiency)
DOCK8 deficiency	STAT3- GOF	DGS
DOCK2 deficiency	Severe congenital neutropenia	IKBA deficiency
IPEX	ADA2 deficiency	NEMO deficiency
WAS	CIQ deficiency	
WIP deficiency	CD25 deficiency	
ARPC1B deficiency	IL-10 deficiency	
CD40 ligand deficiency	IL-10 Receptor deficiency	
CD40 deficiency	DNA double-strand break repair disorders	
XLP1, XLP2		
APDS		
MHC Class II deficiency		
AD Hyper IgE syndrome		
CTLA4 haploinsufficiency		
LRBA deficiency		
Familial HLH types 1–5		
GATA2 deficiency		
RAB27A deficiency		
LAD I		
Reticular Dysgenesis		

∧ *Depending on the clinical and immunological phenotype. SCID, severe combined immunodeficiency; CID, combined immunodeficiency; CGD; chronic granulomatous disease; DOCK8, dedicator of cytokinesis 8; DOCK2, dedicator of cytokinesis 2; IPEX, immune dysregulation, polyendocrinopathy, enteropathy, X-linked; WAS, Wiskott-Aldrich syndrome; WIP, WASP interacting protein; ARPC1B, actin related protein 2/3 complex subunit 1B; XLP1, X-linked lymphoproliferative disease 1; XLP2, X-linked lymphoproliferative disease 2; APDS, activated PI3K delta syndrome; MHC, major histocompatibility complex; AD, autosomic dominant; CTLA-4, cytotoxic T-lymphocyte-associated protein 4; LRBA, lipopolysaccharide (LPS)-Responsive and Beige-like Anchor protein; HLH, hemophagocytic lymphohistiocytosis; GATA2, GATA binding protein 2; RAB27A, member RAS oncogene family; LAD, leukocyte adhesion deficiency; PGM3, phosphoacetylglucosamine mutase; STAT1, signal transducer and activator of transcription 1; STAT2, signal transducer and activator of transcription 2; GOF, gain of function; ADA2, adenosine deaminase 2; CVID, common variable immune deficiency; DGS, DiGeorge syndrome; NEMO, nuclear factor-kappa B essential modulator*.

The development of alternative **donor strategies** and novel graft manipulation techniques has also dramatically improved access to allogeneic HSCT. The different donor sources used in HSCT for PID are shown in Table 3. Donor selection criteria must consider that many patients with PID come from families with a high degree of consanguinity (9), with the risk that matched related donor (MRD)—the gold standard for HSCT—could be genotypically affected, even if still asymptomatic. Although outcomes of HSCT from matched unrelated donors (MUD) are equivalent to those from matched siblings (10), many patients come from ethnic minorities, that are underrepresented in donor registries. But even when no HLA-matched (related or unrelated) donor can be found, valid alternatives are now available. Key studies demonstrated remarkable survival in PID cohorts, using haplo-identical transplants with selective *ex vivo* depletion of αβ T lymphocytes, which are directly involved in graft versus host disease (GVHD), and B cells, which may harbor Epstein–Barr virus (EBV), thus preserving passive innate immunity from the donor and allowing for a faster time to full immune reconstitution (11–14). Re-infusion of genetically modified αβ T lymphocyte receptor bearing cells with an added caspase suicide gene represents a recent modification to the technique; if acute GVHD occurs, the administration of rimiducid, a compound which activates the suicide gene, is able to remove the targeted cells (15). Moreover, a different strategy currently under investigation consists in the depletion of naive T lymphocytes bearing the CD45RA marker, classically implicated in acute GVHD, thus retaining the CD45RO-bearing T cells which are more likely to contribute for antiviral activity (16).

**Table 3:** Donor stem cell sources in PID HSCT.

**Donor source**	**HSC content**	**Access to donor**	**Engraftment time**	**Rate of GVHD**	**Rate of TRM**	**Advantages**	**Disadvantages**	**Other**
MRD	3–5 ×10^∧^6 CD34+ cells/kg or 300–500 ×10^∧^6 TNC/kg	Rapid	Short	Low	Low	Chance to obtain more cells from donor if needed	<30% patients have MRD available, risk of disease-carrier status	Considered standard approach, primary graft source in most pediatric HSCT
MUD		Slow	Short	Increased	Low		HLA mismatches impact outcome	Probability to find compatible donor between 50 and 80%
TCRαβ/CD19- depleted haploidentical donor		Rapid	Short	Low	Low		High rate of viral infections, high laboratory expertise required, risk of disease-carrier status	Increased access to family donors
Cord blood unit	0.3–0.5 ×10^∧^6 CD34+ cells/kg	Rapid	Long	Low	Increased	Increased donor pool for ethnic minorities	Longer immune reconstitution, limited amount of available CD34+ cells, high rate of viral infections, unable to go back to donor for more cells	Ideal in smaller children where adequate HSC dose could be achieved

*MRD, matched related donor; MUD, matched unrelated donor; TCR, T cell receptor; TNC, total nucleated cells; GVHD, graft versus host disease; TRM, transplant related mortality; HLA, human leukocyte antigen; HSCT, hematopoietic stem cell transplantation; HSC, hematopoietic stem cell*.

Modified **conditioning regimens** contributed to improved HSTC outcome. Table 4 describes the most common conditioning strategies currently used in HSCT for PID (17). Conditioning agents allow for creating space in the bone marrow niche, facilitating donor stem cell engraftment. Inclusion of immunosuppressive agents during conditioning reduces the risk of graft rejection. However, irradiation-based conditioning regimens and alkylating chemotherapy are responsible for significant short-term and long-term transplant-related mortality (TRM) and morbidity. The substitution of cyclophosphamide with fludarabine and the development of pharmacokinetics-based busulfan dosing as well as of treosulfan-based conditioning, have shown decreased toxicity and are yet associated with a satisfying stem cell engraftment (18, 19). An alternative reduced intensity conditioning (RIC) regimen based on melphalan and fludarabine is also associated with reduced TRM risk, but an increased rate of mixed chimerism, especially in the myeloid lineage (20).

**Table 4:** Common conditioning regimens used in PID HSCT, adapted from (17).

**Conditioning regimens**^∧^****	**Drug combination**	**+/–**	**Engrafment rate**	**Toxicity**
Myeloablative Conditioning (MAC)^*^	Bu (90 ± 5 mg × h/L iv) + Flu (160 mg/m^2^)	Alemtuzumab or ATG	High	High
Reduced Intensity Conditioning (RIC)	Bu (60 ±5 mg × h/L iv) + Flu (180 mg/m^2^)	Alemtuzumab or ATG	Variable	Low
	Flu (150 mg/m^2^) + Melphalan (140 mg/m^2^)	Alemtuzumab		
	Treosulphan (42 g/m^2^) + Flu (150 mg/m^2^)°	None or Alemtuzumab		

∧ Thiotepa can be used in addition to current conditioning regimen when more myelosuppression is needed.

* Bu/CY conditioning is associated with increased risk of VOD and no longer recommended.

° *Regimen also assessed as reduced toxicity conditioning. Bu, busulfan; Flu, fludarabine; ATG, antithymocyte globulin; CY, cyclophosphamide; VOD, veno-occlusive disease*.

GVHD is primarily due to the recognition of host MHC antigens by donor's naive T cells. Different methods of **GVHD prophylaxis** are outlined in Table 5. Standard GVHD prophylaxis consists of cyclosporine A or tacrolimus in combination with methotrexate or mycophenolate mofetil. Moreover, serotherapy with antithymocyte globulin (ATG) or the anti-CD52 antibody alemtuzumab (Campath) can be added to the conditioning regimen in order to obtain *in vivo* T-cell depletion (21, 22). *Ex vivo* T-cell depletion has been discussed above (see donor strategies). An alternative approach to prevent GVHD in patients receiving T-cell replete mismatched transplant is the use of cyclophosphamide after graft infusion. Cyclophosphamide is selectively toxic to recently activated donor lymphocytes, while preserving donor pluripotent hematopoietic stem cells (23). Recently, Neven et al. demonstrated the efficacy of this approach for patients with life-threatening inherited diseases (24). Several new therapies for steroid-resistant or steroid-dependent acute GVHD are currently under investigation (25); further studies are required for all these new strategies, and for some of them their role in the treatment of acute GVHD has yet to be clearly defined.

**Table 5:** Methods of GVHD prophylaxis and therapy.

**I. Methods of GVHD prophylaxis**
a. Pharmacotherapy
• Calcineurin inhibitor (Tacrolimus^∧^, Cyclosporin A^*^)
• Inhibitors of cell proliferation (Mycophenolate Mofetil, Methotrexate^*^^∧^, PT-Cyclophosphamide)
• Corticosteroids
• mTOR inhibitors (e.g., Sirolimus, Everolimus)
b. Depletion of donor T-lymphocytes
•*In vivo* (Anti-T-lymphocyte globulin^~^, Alemtuzumab)
•*Ex vivo* (CD34+ selection, CD3+ TCRαβ/CD19 depletion, CD3+ TCRαβ/CD19 depletion with iCasp9 suicide gene TCRαβ add-back, CD3+ CD45RA+ depletion)
**II. GVHD Therapies**
a. Corticosteroids
b. Immunosuppressive agents
c. Cytokine-receptor agonists
• Anti-interleukin-2R
• Anti-interleukin-6R
• Anti-TNFR
d. Extracorporeal photopheresis
• 8-Methoxypsor-alen (8-MOP) + UVA radiation
e. Mesenchymal stromal cells
**III. New potential approaches under investigation**
• PI3K inhibitors
• JAK inhibitors
• MEK inhibitors
• Aurora A inhibitors
• ROCK-1 inhibitors
• CDK2 inhibitors

* *In Combination, Europe gold standard*.

∧ *In Combination, America gold standard*.

~ *In Combination. GVHD, graft versus host disease; PT, Post-Transplant; TCR, T Cell Receptor; TNFR, Tumor Necrosis Factor Receptor; PI3K, Phosphoinositide 3-kinase; JAK, Janus kinase; MEK, mitogen-activated protein kinase kinase; ROCK-1, Rho-associated kinase 1; CDK2, Cyclin-dependent kinase 2*.

Finally, the **control of viral infections** after HSCT is fundamental for a good outcome. The development of T lymphocytes specifically directed against viral epitopes represents a powerful strategy to control infections through transplantation. However, at present, only few specific viruses can be targeted and donor banks of virus-specific or multi-virus specific T lymphocytes are available only in specialized centers. Naik et al. showed 81% partial or complete responses against targeted viruses (26). Ongoing clinical trials will define the state of the art of this approach.

## Hematopoietic Stem cell Transplantation for Individual Primary Immunodeficiency Diseases

The broad spectrum of clinical and immunological phenotypes associated with PID makes it difficult to define a universal transplant regimen. Here we describe the evidence regarding the different approaches to HSCT for individual PID. Table 6 shows an overview for each PID analyzed in the text.

**Table 6:** Overview of HSCT in individual PID.

**Indication**	**Main PID features**	**Evidence summary**	**Level of evidence based on published patients (<10; 10–100; >100)**
X-linked SCID and JAK3 deficiency; IL7R deficiency	Impaired γc signaling resulting in SCID T– B+ NK–; Impaired IL7R signaling resulting in SCID T- B+ NK+	HSCT required for survival. Conditioning is not required to attain T cell reconstitution. However, in the absence of conditioning, functional B and NK cell reconstitution is typically not achieved in X-linked SCID and JAK3 deficiency. In IL7R deficiency B and NK cells are functional, thus no or low-dose conditioning is indicated	>100 (X-linked SCID and JAK3 deficiency); 10–100 (IL7R deficiency)
RAG deficiency	Impaired VDJ recombination, leading to defective T and B cell development. Clinical presentation: autosomal recessive T- B- NK+ SCID, Omenn syndrome, atypical SCID, combined immune deficiency with granulomas and/or autoimmunity (CID-G/AI)	Patients with SCID and Omenn: HSCT required for survival. Use of RIC was associated with better T and B cell reconstitution. Patients with CID-G/A: HSCT + conditioning should be considered early in the course of the disease	>100 (SCID, Omenn and leaky SCID); 10–100 (CID-G/AI)
Adenosine deaminase (ADA) deficiency	Metabolic disease that may affect different tissues and organs; decreased cell survival	HSCT is curative. Gene therapy is an alternative option. ERT can be used as bridge to HSCT or gene therapy. Survival is superior after unconditioned HSCT than after MAC or RIC	10–100
Reticular Dysgenesis	T- B- NK- SCID, agranulocytosis, and sensorineural deafness due to mutations in *AK2* gene	HSCT required for survival. Myeloablative components in the conditioning regimens required to achieve high-level donor myeloid engraftment and avoid post-transplant neutropenia	10–100
DNA double-strand break repair disorders	Heightened sensitivity to ionizing radiation due to defects in components of the non-homologous end joining (NHEJ) DNA repair mechanism	Associated immunodeficiency can be resolved by HSCT. Increased short- and long-term sensitivity to the alkylator-based conditioning regimens. Better survival with RIC than MAC	10–100
MHC class II (MHC-II) deficiency	Lack of MHC-II expression is associated with low CD4+ cell count, impaired antibody production, defective T cell priming	Without successful HSCT, most patients succumb in the first decade of life. Indication to HSCT depends on clinical status of the patient and availability of a matched donor, but survival is lower than in other forms of PID	>100
CD40 ligand and CD40 deficiency	Defective CD40 signaling, leading to impaired immunoglobulin class switch and defective dendritic cell activation and T cell priming	HSCT is curative. Event-free survival: best with MAC and absence of pre-existing organ damage (in particular sclerosing cholangitis)	>100
DOCK8 deficiency	Deficiency in DOCK8 is responsible for abnormal cytoskeletal rearrangement. Patients present with severe eczema, immunodeficiency, autoimmunity, severe allergies and increased risk for malignancy	HSCT curative, best outcome with RIC	10–100
DOCK2 deficiency	Deficiency in DOCK2 lead to early-onset severe bacterial and viral infections with T cell lymphopenia, reduced naïve T cells, defective antibody responses and impaired NK cell function	HSCT curative, no conclusive data regarding preferred conditioning regimens	<10
Functional T cell immunodeficiencies	Defective pre-TCR and TCR signaling	HSCT is required for survival in patients with CD3δ, CD3ε, or CD3ζ defects. CD3γ deficiency may only require HSCT in most severe cases. There is limited experience in other TCR signaling defects. Overall, conditioning is beneficial to achieve immune reconstitution, but its intensity must be tailored to minimize risks of organ toxicity	10–100
Wiskott-Aldrich Syndrome and other immunodeficiencies with thrombocytopenia (WIP, ARPC1B)	WAS: X-linked disorder with immunodeficiency, eczema and thrombocytopenia. WIP: autosomal recessive immunodeficiency with mutations in the *WIPF1* gene. Patients display a WAS-like phenotype. ARPC1B: autosomal recessive CID with immune dysregulation and platelet abnormalities	HSCT curative. Low myeloid engraftment is associated with increased risk of persistent thrombocytopenia. High intensity conditioning regimens result in reliable donor chimerism	>100 (WAS); <10 (WIP); <10 (ARPC1B)
Cartilage hair hypoplasia (CHH)	Caused by mutations of the *RMRP* gene involved in ribosomal RNA processing, mitochondrial DNA replication and control of gene transcription. Syndromic combined immunodeficiency, with short stature, sparse hair, and increased risk of autoimmunity, Hirschsprung disease, bone marrow failure and malignancies	HSCT can cure the immune deficiency and help prevent infections, bone marrow failure and malignancy. However, growth, cutaneous and intestinal manifestations of the disease are not cured	10–100
AD hyper-IgE syndrome due to dominant negative *STAT3* mutations (Job's syndrome)	Mutation in the *STAT3* gene. The disease is characterized by hyper-IgE, increased occurrence of bacterial and Aspergillus infections, mucocutaneous candidiasis, somatic features (distinctive facies, scoliosis, coronary and cerebral aneurysms) and deficiency of Th17 and T follicular helper cells	There is inconclusive evidence that HSCT is beneficial, although clinical and immunological improvement has been reported in several cases. However, the impact of transplantation on other features (aneurysms, bone anomalies and possibly intrinsic lung abnormalities) is not clear	10–100
Phosphoglucomutase 3 (PGM3) deficiency	Glycosylation defect presenting with variable immunodeficiency, skeletal dysplasia, neurodevelopmental delay, tendency to bone marrow failure and organ (kidney, intestine, heart) defects	HSCT may be curative, but limited experience. Other manifestations of the disease: unlikely to be resolved by HSCT. Intermediate intensity conditioning recommended	<10
Chronic granulomatous disease (CGD)	Mutations that affect the functionality of the nicotinamide adenine dinucleotide phosphate (NADPH) complex, with defective production of microbicidal reactive oxygen species. The most common form is X-linked	HSCT curative. Adequate level of donor myeloid chimerism is fundamental to successfully correct the clinical phenotype. To limit toxicity, alkylator-based RIC are used	>100
Immuno-dysregulation, Polyendocrinopathy, Enteropathy, X-Linked (IPEX syndrome)	Mutations in the *FOXP3* gene, a master transcriptional regulator for development of CD4 regulatory T-cells. Patients experience severe, multi-organ autoimmune phenomena including enteropathy, chronic dermatitis, endocrinopathy, hepatitis, nephritis and cytopenia	Patients do not survive long-term without HSCT, HSCT curative. Important to transplant before organ damage develops. Medium-high RIC regimen may suffice to correct the disease, unclear whether serotherapy is needed	10–100
Activated PI3kinase delta syndrome (APDS)	GOF mutations of *PIK3CD* or LOF mutations of the *PIK3R1* gene, encoding the regulatory subunit p85a. Patients manifest defects in T-cell function with deficiency of naive T cells and an excess of senescent effector T cells, defects in B-cell function with increased IgM, reduced IgG2, and impaired vaccine responses, recurrent sinopulmonary infections, lymphoproliferation	HSCT can be curative. Serotherapy may help prevent graft failure/rejection. Medium/high RIC regimens are associated with improved chimerism and immune function	10 - 100
STAT1 GOF	*STAT-1* GOF mutations impair STAT1 dephosphorylation or result in increased STAT1 phosphorylation. Patients experience chronic mucocutaneous candidiasis, severe viral infections, bacterial and mycobacterial infections, and autoimmunity	There is need to improve approach to HSCT for this disease. A sub-myeloablative regimen may be needed. The role of serotherapy has to be evaluated	10–100
STAT3 GOF	Mutations confer GOF in STAT3 leading to secondary defects in STAT5 and STAT1 phosphorylation and the regulatory T-cell compartment. Patients experience infections, lymphoproliferation and autoimmunity	There is very limited experience with HSCT for this disease. HSCT can be curative, and RIC with serotherapy may suffice	<10
CTLA-4 deficiency	Heterozygous mutations in *CTLA4* leading to haploinsufficiency and impaired CTLA4 dimerization or impaired ligand binding, result in an autosomal dominant immune dysregulation syndrome with immunodeficiency	There is very limited experience with HSCT for this disease. HSCT can be curative, and RIC with serotherapy may suffice	<10
LRBA deficiency	Immune dysregulation syndrome due to mutations in LPS-responsive, beige-like anchor (*LRBA*), resulting in hypogammaglobulinemia with B-cell deficiency, functional T-cell defects, aberrant autophagy, autoimmunity and chronic diarrhea	There is very limited experience with HSCT for this disease. HSCT can be curative	<10
X-linked lymphoproliferative disease (XLP) 1 and 2	XLP1 (due to mutations of the *SH2D1A* gene) affects cytotoxic T and NK cell function. Affected males are at high risk for fulminant infectious mononucleosis, HLH, EBV-driven lymphoma, bone marrow aplasia, and hypogammaglobulinemia. XLP2 (with mutations of *XIAP*) is characterized by increased incidence of HLH and inflammatory disease (especially of the gut), but not of EBV-related lymphoma	HSCT can be curative. RIC is preferable to MAC. There is a need to improve outcome for patients with XLP2, and these patients may continue to experience inflammatory intestinal disease even after successful transplant	>100 (XLP1); 10–100 (XLP2)

*PID, primary immunodeficiency; HSCT, hematopoietic stem cell transplantation; SCID, severe combined immunodeficiency; CID, combined immunodeficiency; JAK3, Janus kinase 3; IL7R, interleukin-7 receptor; RAG, recombination activating gene; CD40, cluster of differentiation 40; DOCK8, dedicator of cytokinesis 8; DOCK2, dedicator of cytokinesis 2; WAS, Wiskott-Aldrich syndrome; WIP, WASP interacting protein; ARPC1B, actin related protein 2/3 complex subunit 1B; AD, autosomal dominant; STAT3, signal transducer and activator of transcription 3; GOF, gain of function; STAT1, signal transducer and activator of transcription 1; CTLA-4, cytotoxic T-lymphocyte-associated protein 4; LRBA, lipopolysaccharide (LPS)-responsive and beige-like anchor protein; AK2, adenylate kinase 2; TCR, T-cell receptor; RMRP, RNA component of mitochondrial RNA processing endoribonuclease; FOXP3, Forkhead box P3; LOF, loss of function; PIK3CD, phosphatidylinositol-4,5-bisphosphate 3-kinase catalytic subunit delta; PIK3R1, phosphoinositide-3-kinase regulatory subunit 1; STAT5, signal transducer and activator of transcription 5; SH2D1A, SH2 domain containing 1A; HLH, hemophagocytic lymphohistiocytosis; EBV, Epstein-Barr virus; RIC, reduced intensity conditioning; ERT, enzyme replacement therapy; MAC, myeloablative conditioning*.

The literature review has been performed employing PubMed, EMBASE, Web of Science, and Scopus databases, retrieving all publications on the outcome of HSCT for individual PID. We performed the search strategy using a free-text search (keywords) and thesaurus descriptors search (MeSH and Emtree) for each individual PID, adapted for all the selected databases. We searched all articles published up to May 2019. Articles were considered eligible according to the following inclusion criteria: English language and publication in peer-reviewed journals. Articles were excluded by title, abstract, or full text for irrelevance to the investigated issue. Lastly, to identify further studies that met the inclusion criteria, the references of the selected articles were also reviewed.

### X-Linked SCID, JAK3 Deficiency, and IL-7 Receptor Deficiency

**X-linked SCID (X-SCID)** is a X-linked recessive inherited immunodeficiency that presents with the clinical features of SCID in the first few months of life, i.e., failure to thrive, chronic diarrhea, recurrent respiratory infection, and/or generalized infections from opportunistic pathogens, or signs of graft versus host reaction (skin rash, pancytopenia, abnormalities of liver function) from transplacental acquired maternal T cells. The immunological phenotype is characterized by the lack of peripheral blood T and NK lymphocytes; B cells are usually present, but immunoglobulin production is severely reduced, if not absent. X-SCID is caused by mutations in the interleukin-2 receptor gamma (IL2RG) gene encoding for the common gamma chain (γc) shared by cytokine receptors for interleukin (IL)-2, IL-4, IL-7, IL-9, IL-15, and IL-21. The clinical and immunological phenotype of X-SCID is virtually undistinguishable from that of **JAK3 (Janus Kinase 3) deficiency**. JAK3 is coupled with the common γ chain shared by the above cited cytokine receptors and mediates their downstream signaling; its deficiency is responsible for the autosomal recessive T– B+ NK– SCID.

Buckley et al. reported on a large cohort of patients with SCID receiving HSCT, including 89 infants transplanted at Duke between 1992 and 1998 (27).

The majority of patients with SCID were boys with X- SCID. However, six had *JAK3* mutations, 13 had adenosine deaminase deficiency (ADA)-SCID, three had IL-7 receptor α chain defects, and 21 infants had unclassified autosomal recessive SCID.

A total of 77 infants received T cell depleted, haploidentical parental HSCT, and 12 received a MRD transplant. The survival rate was just over 80 percent 3 months to 16.5 years post-transplant. None of these patients received chemotherapy pre-transplant or GVHD prophylaxis. GVHD developed in 28 of the 77 infants who received T cell depleted, HLA haploidentical marrow, but in most cases the GVHD was mild and did not require treatment. Interestingly, B cell function was reconstituted in <50% of the long-term survivors necessitating life-long immunoglobulin replacement in those patients. It has been demonstrated that signaling through the common γ-chain and JAK3 is required to enable B cells to respond to IL-21 and differentiate into plasmablasts (28). In the absence of donor B cell engraftment, B lymphocytes from patients with X-SCID and JAK3 deficiency remain unable to produce antibodies, even when robust donor T cell engraftment is achieved (29). Furthermore, waning of T cell immunity has been observed at long-term follow-up in several patients with X-SCID and JAK3 deficiency who did not receive conditioning chemotherapy at the time of HSCT. These data argue in favor of including some chemotherapy in the conditioning regimen for these patients, if clinical conditions allow, in order to enable durable stem cell engraftment and full T and B cell immune reconstitution.

Hamid et al. reported an overall survival of 72% following HSCT of 43 patients with IL2RG/JAK3 SCID (30). Sixty-eight percent of them had ongoing medical issues. B-lymphoid and myeloid chimerism after HSCT were highly correlated. Low-toxicity myeloablative conditioning (MAC) resulted in better B-lymphoid/myeloid chimerisms and freedom from IVIG replacement. The authors concluded that a dose of busulfan of 8 mg/kg in combination with cyclophosphamide might not be myeloablative enough to ensure a robust donor HSC engraftment with donor B-lymphopoiesis. Moreover, the authors speculate that in conditioned recipients the thymic niche is consistently reseeded from the bone marrow-derived donor stem cells leading to ongoing thymopoiesis, while for unconditioned patients, initial seeding of the thymic niche by infused HSC is not generally followed by reseeding, as donor stem cell engraftment does not consistently occur in the bone marrow, and thymic seeding may have a finite lifetime, leading eventually to thymic exhaustion.

**IL-7 Receptor Deficiency** is an autosomal recessive form of SCID, with selective lack of T cells, but normal number of B and NK lymphocytes (31). At variance with X-linked SCID and JAK3 deficiency, where B cells are genetically impaired in their capacity to respond to IL-21 and differentiate into antibody-secreting cells, IL7R-deficient B cells are functional, as long as T cell help is provided. This observation has represented the rationale for performing HSCT in the absence of conditioning for IL7R-deficient patients. Dvorak et al. have reported the cases of two infants with SCID due to IL7R deficiency, associated with maternal T cell engraftment in one of them (32). The patients received HSCT from an unrelated donor with conditioning consisting of alemtuzumab only, which temporarily ablated circulating T cells in the patient with maternal engraftment. Both patients showed slow, but sustained T cell engraftment in the absence of myeloid or B cell donor chimerism. However, reconstitution of donor-derived T cells was sufficient to enable also humoral immune reconstitution. These observations confirm that chemotherapy is not strictly required to induce both cellular and humoral immune reconstitution in patients with IL7R deficiency. However, it remains to be seen whether in the absence of donor stem cell chimerism, robust thymic T cell output will be maintained over time.

### RAG Deficiency

The Recombination Activating Genes 1 and 2 (*RAG1, RAG2*) assemble as a heterotetramer that initiates the process of V(D)J recombination in developing T and B cells, allowing for the assembly of T cell receptor and immunoglobulin molecules, thus for T and B cell development (33). Null mutations in *RAG1* or *RAG2* cause autosomal recessive **T– B– NK+ SCID** in humans, however hypomorphic mutations in the same genes have been associated with a spectrum of clinical phenotypes, including **Omenn syndrome**, **atypical SCID** and **combined immune deficiency with granulomas and/or autoimmunity (CID-G/AI)** (33). If untreated, severe RAG deficiency is inevitably fatal early in life; furthermore, although CID-G/AI may allow survival into late childhood or even adulthood, these patients are at high risk of severe organ damage (especially in the lungs), chronic and severe herpes virus infections, and severe autoimmune manifestations, ultimately leading to reduced survival and poor quality of life (34–36).

For several decades, the results of HSCT for RAG deficiency had been less satisfactory than in other forms of SCID or CID, especially when no matched related donors were available. A multicenter study reported outcome of HSCT in 76 patients with RAG deficiency (37). In this study, survival was ~80% for 25 patients who received unmanipulated bone marrow graft from matched related donors without myeloablative conditioning. However, while T cell reconstitution was achieved, the CD4+ T cell count remained in the lower range of normal in the majority of the patients, and reconstitution of B cell immunity was observed less consistently, with more than half of the patients requiring regular immunoglobulin replacement therapy. In the same study, 39 patients received T-cell-depleted transplantation from haploidentical donors. Among those who received transplantation without chemotherapy or with cyclophosphamide or fludarabine only (with variably added serotherapy), only 25% of them engrafted and developed sustained T-cell immunity, and none developed B cells. Finally, survival was 60% among RAG-mutated patients who received T-cell-depleted HSCT from a haploidentical donor with chemotherapy conditioning (mostly with busulfan-based regimens); the majority (82%) of these patients attained T and B cell reconstitution, but 18% required immunoglobulin replacement therapy. There was an association between use of chemotherapy, stem cell engraftment and B cell reconstitution. A recent retrospective study from the Primary Immune Deficiency Treatment Consortium (PIDTC) reported on the outcome of 662 patients with SCID and related diseases who received HSCT between 1982 and 2012, including 52 patients with RAG deficiency (38). Among these, 28 presented with SCID, and 24 with atypical SCID or Omenn syndrome. Thirty-nine of these patients received haploidentical HSCT, with no difference in survival observed between those with SCID and those with atypical SCID/Omenn syndrome. Overall survival for patients with RAG deficiency was comparable to that observed for patients with X-linked SCID and JAK3 deficiency and use of conditioning regimen was not identified as an independent contributor to overall survival. On the other hand, genotype, donor type and conditioning regimen were found to affect the quality of immune reconstitution. Patients with RAG deficiency experienced poorer T- and B-cell reconstitution after HSCT. Moreover, recipients of haploidentical transplantation attained poorer B-cell reconstitution. Finally, use of reduced intensity conditioning or myeloablative conditioning was associated with better T-and B-cell reconstitution than no conditioning or immunosuppression only. More limited data are available on HSCT in patients with CID-G/AI (36). Twenty-six patients with RAG deficiency presenting as CID-G/AI were transplanted at a mean age of 5.2 years (35). In most cases, myeloablative conditioning was used. At a median follow-up on 9 months, 18 patients (69.2%) were reported to be alive. As many of these patients have been diagnosed late in the course of the disease, when organ damage is already often manifest, it is believed that transplant should be offered sooner, and conditioning regimen should be tailored to facilitate stem cell engraftment, deplete dysreactive T- and B-cell clones, while minimizing the risks of organ toxicity.

### Adenosine Deaminase (ADA) Deficiency

Adenosine deaminase (ADA) is an enzyme involved in purine metabolism. ADA deficiency is responsible for a severe immunodeficiency affecting all lymphoid lineages (T– B– NK– SCID). Clinical manifestations of the disease include severe infections since early in life, neurodevelopmental delay, hearing defects, liver dysfunction, pulmonary alveolar proteinosis, skeletal defects, increased risk of tumors (lymphoma, dermatofibrosarcoma protuberans, and liver cancer), and autoimmune manifestations (39). Most patients die early in life unless treated by enzyme replacement therapy (ERT), HSCT or gene therapy. In particular, use of ERT has been shown to be efficacious in improving immune function, hepatocellular abnormalities, pulmonary alveolar proteinosis, and bone dysplasia. However, several patients have experienced deterioration in lymphocyte counts and function over time, which may contribute to reappearance of a higher risk of infection and tumors. For these reasons, ERT is often considered as a bridge treatment while prompting for HSCT or gene therapy. A multicenter study included 54 patients with ADA-SCID who have received HSCT from MRD, 15 patients who received HSCT from MUD, 7 patient who were transplanted from mismatched unrelated donors, and 30 from a haploidentical donor (40). Overall survival was higher for patients who received HSCT from MRD; within this group there were 46 (85.2%) survivors, with 3 (5.6%) patients dying from treatment-related causes. Furthermore, conditioning was not necessary for patients receiving HSCT from MRD, as donor chimerism was reported in 100% of those who did not receive conditioning and in 80% of those who did. Overall survival in patients who received HSCT from MUD was 67%. Lower survival rates were recorded for patients who received a mismatched unrelated donor transplantation or a haploidentical transplant (29 and 43% overall survival rates, respectively). In these group of patients most of the death were observed in the first 100 days after transplant, and were mainly due to infections. When considering the role of conditioning, improved overall survival was observed in unconditioned transplantations (78%) as compared to patients who received myeloablative or reduced intensity conditioning (56 and 67% overall survival rates, respectively). Recently, a single center-experience with unconditioned MRD transplantation for ADA-SCID showed that 4 of 16 patients required a repeat procedure (39). These data suggest that if ERT is used to induce immune reconstitution, it should be discontinued before HSCT; alternatively, mild conditioning should be used to deplete cells generated while receiving ERT.

### Reticular Dysgenesis (RD)

Mutations in the gene encoding adenylate kinase 2 (*AK2*) are responsible for reticular dysgenesis (RD), clinically defined by the combination of T– B– NK– SCID, agranulocytosis, and sensorineural deafness. Hoenig et al. reported on clinical presentation, genetics, and treatment outcome for a total of 32 patients born between 1982 and 2011 (41). Thirty-one patients received HSCT (one patient died for neonatal sepsis before HSCT). Grafts derived from mismatched family donors (*n* = 17, 55%), MRD (*n* = 6, 19%), and unrelated marrow or umbilical cord blood donors (*n* = 8, 26%). Secondary or tertiary transplants were required in 13 patients. At a mean follow-up of 7.9 years after transplantation, 68% of patients were reported alive. Interestingly, persistent or recurrent agranulocytosis due to failure of donor myeloid engraftment has been reported in all patients who died beyond 6 months after HSCT. Transplant without conditioning resulted ineffective to overcome agranulocytosis. In comparison with other PID, although long-term survival is possible in the presence of mixed chimerism, inclusion of myeloablative components in the conditioning regimens was needed in order to achieve high-level donor myeloid engraftment and avoid post-transplant neutropenia.

### DNA Double-Strand Break Repair Disorders

Inherited defects in components of the non-homologous end joining (NHEJ) DNA repair mechanism are responsible for heightened sensitivity to ionizing radiation. Patients with these disorders have also increased short- and long-term sensitivity to the alkylator-based conditioning regimens. Known causes of radiosensitive T– B– NK+ SCID include deficiencies of **Artemis**, **DNA Ligase IV**, **DNA-dependent protein kinase catalytic subunit (DNA-PKcs)**, and **Cernunnos–XRCC4-like factor (Cernunnos-XLF)** (42), while **ataxia-telangiectasia (AT)** and **Nijmegen breakage syndrome** are counted among non-SCID forms of radiosensitive immunodeficiencies. Schuetz et al. showed that the use of alkylating agents in Artemis deficiency, although not affecting overall survival -that resulted equivalent to that observed in patients with RAG-deficient SCID-, was responsible for long-term sequelae on growth and development due to the effect of chemotherapy on mutated somatic cells (37). Slack et al. analyzed HSCT outcome data for a total of 87 patients with DNA ligase IV deficiency (*n* = 36), Cernunnos-XLF deficiency (*n* = 17), Nijmegen breakage syndrome (*n* = 26), and AT (*n* = 8) (43). Survival of patients who received conditioning was 69% and was worse for MAC than for RIC. Moreover, GVHD was more frequent in patients receiving MAC compared with those receiving RIC (57 vs. 46%). Most deaths occurred early after conditioning, suggesting reduced tolerance to chemotherapy. Survival in patients with AT was only 25%. When the transplant was successful, while immune-mediated complications resolved, growth and developmental delay remained after HCT.

### Major Histocompatibility Complex (MHC) Class II Deficiency

MHC class II deficiency comprises a genetically heterogeneous group of autosomal recessive disorders due to mutations in transcription factors that drive expression of MHC class II molecules. Without successful HSCT, most patients with MHC class II deficiency succumb to infections in the first decade of life. The immunological hallmarks of the disease include: lack of HLA class II antigen expression, markedly reduced count of CD4+ T cells, and lack of antigen-specific responses (44). The indication to HSCT depends on clinical status of the patient and availability of a matched donor. European Registry data show that survival after HSCT for MHC class II deficiency is lower than in other forms of combined immunodeficiency (45).

Siepermann et al reviewed the outcome of 76 transplants in 68 patients with this disease (46). MRD was available in 56 cases, a partially matched related donor in 12 and MUD in eight. In 41 patients, a myeloablative conditioning was used, mostly with busulfan and cyclophosphamide in combination with T-cell depleting serotherapy. Bone marrow was used as stem cell source in 66 patients. Graft rejection was documented in 10 patients. Six patients developed grade III or IV GVHD, with a high occurrence of concurrent viral infections. Overall, there was a high mortality rate, with 42 deaths, 4 of which in the pre-transplant phase and 38 post-transplant. The death rate by donor type was as follows: MRD, 25 deaths/56 transplants; partially matched related donors, 8/12; MUD, 5/8. The main causes of death were viral, bacterial or fungal infections, often associated with GVHD. Because HSCT would not correct impaired MHC class II expression on thymic epithelial cells and other stromal cells, thymic selection may result in altered function and phenotype of T cells post-transplant, which may in turn cause delayed and incomplete immune reconstitution, and increased risk of infections.

Small et al. reported on the outcome of HSCT in 16 consecutive cases of MHC class II deficiency treated at Memorial Sloan Kettering, New York (47). Eight of the patients had required mechanical ventilation pre-transplant. Donors included HLA-mismatched family members in 10 cases, MUD in 4, and unrelated cord blood in 2. Seven patients received a T cell-depleted transplant, six of whom were treated with busulfan (16 mg/kg), combined with thiotepa and fludarabine (*n* = 3), thiotepa and cyclophosphamide (*n* = 1), cyclophosphamide and fludarabine (*n* = 1), or cyclophosphamide alone (*n* = 1). All patients who received T-cell depleted transplants also received ATG or alemtuzumab. Recipients of T cell-replete transplant were treated with busulfan, cyclophosphamide and fludarabine (*n* = 2), thiotepa and cyclophosphamide (*n* = 1), melphalan, fludarabine and alemtuzumab (*n* = 2), fludarabine, cyclophosphamide, anti-CD45 and alemtuzumab (*n* = 1), or low dose cyclophosphamide, fludarabine, 200 cGy total body irradiation (TBI) and post-transplant cyclophosphamide. Graft failure occurred in 5 patients, all of which received a second transplant. Eleven (69%) patients survived without GVHD at a median follow-up of 5.7 years. CD4+ T cell reconstitution was suboptimal and declined progressively over time, suggesting defective thymopoiesis.

Elfeky et al. have reported on the use of unrelated cord blood transplantation without serotherapy in 6 patients with MHC class II deficiency, 5 of whom had chronic norovirus infection (48). Other active infections at the time of transplant included: CMV viremia, *Pneumocystis jiroveci* pneumonia, Echovirus meningitis, and RSV pneumonitis. Conditioning was with treosulfan (42 g/m^2^) and fludarabine (150 mg/m^2^). All patients attained >90% donor chimerism by 28 days, followed by stable mixed chimerism of >60% in all patients except one. Patients had good reconstitution of total CD3+ and of CD4+ counts. Four patients were able to clear the Norovirus infection. However, four patients remained dependent on IVIG replacement, one patient developed autoimmune thrombocytopenia and pulmonary hemorrhage in the presence of HHV6 pneumonitis and one patient suffered from grade III GVHD.

### CD40 Ligand and CD40 Deficiencies

CD40 ligand (CD40L) is a cell surface molecule that is predominantly expressed by activated CD4+ T cells and plays a critical role in stimulating B cell maturation and plasma blast differentiation during the germinal center reaction, as well as in inducing dendritic cell and monocyte/macrophage activation by interacting with its receptor CD40 (49).

**CD40L deficiency** in humans causes X-linked hyper-IgM syndrome, a condition characterized by recurrent infections sustained by bacteria, viruses, and opportunistic pathogens (including *Cryptosporidium, Pneumocystis jiroveci*, and CMV) and neutropenia. The immunological phenotype includes hypogammaglobulinemia with normal to elevated levels of IgM, and markedly reduced number of switched memory B cells. Although the clinical phenotype may have variable severity, many patients—if left untreated—succumb early in life to infections, and even with immunoglobulin replacement therapy and antimicrobial prophylaxis there is an increased risk of severe biliary tract disease and liver failure, especially secondary to Cryptosporidium infection, and of peripheral neuroectodermal tumors (50).

Thomas et al. were the first to report in 1995 successful correction of CD40L deficiency by allogeneic HSCT (51).

In 2004, Gennery et al. reported on the European experience with HSCT in 38 patients with this disease (52). Sixteen patients had a past history of *P. jiroveci* pneumonia, and 19 patients had experienced Cryptosporidium infection. Twenty patients had abnormal liver function at the time of transplant, and 15 of them had evidence of sclerosing cholangitis at liver biopsy. Donors included 14 MRD, 22 unrelated donors (15 of which were fully matched to the recipient), and 2 phenotypically matched parents. Twenty-nine patients received myeloablative conditioning with busulfan (12–20 mg/kg) and cyclophosphamide (200 mg/kg), with the addition of alemtuzumab or thiotepa in one patient each. Nine patients received RIC, which included fludarabine and melphalan in 8 patients, 5 of which also received ATG; one patient received TBI with partial hepatic shielding, and cyclophosphamide. Thirty-four patients engrafted with full or mixed chimerism; 4 patients showed autologous reconstitution, and one of them received a second transplant from a different donor with conditioning. Despite the fact that 20 patients had abnormal liver function, veno-occlusive disease (VOD) was seen in only 4 cases. However, GVHD grade II-IV was seen in 14 patients, and was fatal in 6 with pre-existing infection. One patient developed fulminant liver failure and required an orthotopic liver transplantation, but died of disseminate cryptosporidiosis. Of 4 patients who had received liver transplantation prior to HSCT, 3 died. Overall, 22 patients (58%) were cured and expressed CD40L on the cell surface; 20 of these no longer required immunoglobulin replacement therapy. There were 12 deaths, all of which were associated with infections. In particular, *Cryptosporidium* and adenovirus were frequently involved. Pre-existing lung damage was a negative prognostic factor for survival. Furthermore, among patients who received HSCT from an unrelated donor, the outcome was significantly worse for the 7 patients who were transplanted from a 1-Antigen mismatched donor (4 of which died, and 1 showed autologous reconstitution) than among the 15 whose donors were fully matched (with 3 deaths and 2 autologous reconstitutions in these cases).

Jacobsohn et al. reported on the use of non-myeloablative conditioning (fludarabine 180 mg/m^2^; busulfan 6.4 mg/kg; horse ATG 160 mg/kg) in two unrelated patients with CD40L deficiency and cholangiopathy (53). Both patients received HSCT from their HLA-matched siblings. One patient attained full donor chimerism, whereas mixed chimerism (25–45%) was observed in myeloid and lymphoid cells of the other patient. Both immunological and clinical improvement were noted in both patients.

Mitsui-Sekinaka reported on 29 patients with CD40L deficiency who received HSCT in Japan (54). The median age at transplant was 7 years. Donors included: MRD (*n* = 13), MUD (*n* = 13), 6/6 or 5/6 matched unrelated cord blood (*n* = 3). Sixteen patients received myeloablative conditioning with busulfan (16 mg/kg) and cyclophosphamide (200 mg/kg), with the addition of ATG in 2 patients. One patient received TBI (12 Gy) and cyclophosphamide. Ten patients received RIC. Five patients had pre-transplant organ damage; three of them received myeloablative conditioning, and 2 received RIC regimens. The overall survival was 100% at 10 years and 65.9% at 30 years. Among patents without pre-transplant organ damage, full donor chimerism was observed in 14/16 (87.5%) of those who received myeloablative conditioning, and in 4 of 8 (50%) treated with RIC regimens. Twenty of the 25 living patients fully engrafted, and 22 (88%) remained free of IVIG substitution. Graft failure was seen in 3 patients despite myeloablative conditioning. Mixed chimerism was seen in 3 patients, 2 of which had received a RIC regimen. All three continued to require IVIG replacement therapy for more than 1 year after HSCT. Post-transplant complications included pulmonary aspergillosis in 4 patients, *P. jiroveci* pneumonia in 2, *C. parvum* infection in 2, and viral encephalitis in 2.

De la Morena et al. have compiled a recent study of long-term outcome in 176 patients with CD40L deficiency, including 67 who received HSCT (55). The majority (*n* = 47) of the patients received unrelated donor HSCT; 37 patients received HSCT from a matched donor; 17 from a 1-antigen mismatched donor; 2 from 2-antigen-mismatched donor; and 2 from a haploidentical donor. No information of donor/recipient matching was available in 2 cases. Myeloablative conditioning was used in 41 patients, and RIC in 20. No information on type of conditioning was available for 6 patients. Among 65 patients for whom information on engraftment was available, 57 engrafted. In particular, engraftment was achieved in 93% of those who had received myeloablative conditioning, and in 85% of those who had received a RIC regimen. Nine patients required a second HSCT. At the close of the study, 57 (85%) of these 67 patients were surviving. Infections and transplant-related complications (VOD and GVHD) were the main causes of death. Liver disease at the time of transplantation represented a negative risk factor for survival. An improved outcome of HSCT for CD40L deficiency in recent years has been confirmed by Ferrua et al. who reported on 130 patients who received transplantation between 1993 and 2015 (56). In this series, overall survival and event-free survival were 78.2 and 58.1%, respectively. Results were significantly better for transplants performed in year 2000 or later and in patients <10 years old at the time of HSCT. Event-free survival was superior when matched sibling donors, myeloablative conditioning, and bone marrow as a source of stem cells were used. Pre-existing organ damage and sclerosing cholangitis in particular, were negative predictive factors.

**CD40 deficiency** is inherited as an autosomal recessive trait, and its clinical and immunological phenotype mirrors what observed in males with CD40L deficiency (57). The first patient with this disease who received HSCT was an infant with severe respiratory infections, chronic diarrhea, failure to thrive, and disseminated *C. parvum* infection complicated by sclerosing cholangitis (58). She received peripheral blood stem cells (PBSC) transplant from her matched brother, upon RIC regimen with fludarabine (150 mg/m^2^), melphalan (100 mg/m^2^) and rabbit ATG (12.5 mg/kg). The post-transplant course was complicated by severe respiratory infection and distress, leading to death on day +16. Mazzolari et al. were the first to report on successful HSCT for CD40 deficiency (59). The patient was a 3-year-old child with a history of recurrent infections and neutropenia. She received HSCT from her matched sibling (who was heterozygous for the mutation), upon myeloablative conditioning with busulfan (16 mg/kg) and cyclophosphamide (200 mg/kg). Stable multilineage full donor chimerism was observed, associated with correction of the disease phenotype. Al-Saud et al. have reported on HSCT in 6 patients with CD40 deficiency (60). Five patients had a history of respiratory tract infections; two had *C. parvum* infection and sclerosing cholangitis and one patient had CMV viremia. Neutropenia was present in 4 patients. Five of the 6 patients received MRD HSCT, and one was transplanted from a 1-antigen mismatched sibling donor. Myeloablative conditioning with busulfan (16 mg/kg) and cyclophosphamide (200 mg/kg) was used in all patients, one of which also received ATG (10 mg/kg). Complications post-transplant included: drug-induced nephrotoxicity and VOD in one patient each. All patients survived at a median follow-up of 54 months. At the time of last follow-up, mean donor chimerism was 66.6% (range: 16–100%) in lymphocytes, and 75.5% (range: 51–100%) in myeloid cells.

### DOCK8 Deficiency

Dedicator of cytokinesis 8 (DOCK8) deficiency is responsible for abnormal cytoskeletal rearrangement, resulting in defective cell migration and adhesion and abnormal cell structure (61, 62). Severe eczema, immunodeficiency with increased susceptibility to bacterial and viral infections, autoimmunity, severe allergies and increased risk for malignancy represent the main clinical features (63).

Given the poor outcome on conservative treatment, a number of case reports and small series report on transplanted patients (64–72). Recently, Aydin et al. reported on HSCT outcome data for 81 patients from 22 centers transplanted at a median age of 9.7 years (range, 0.7–27.2 years) between 1995 and 2015 (63). After a median follow-up of 26 months, 68 (84%) patients were reported to be alive. Causes of death were infections (*n* = 5), GVHD (*n* = 5), multiorgan failure (*n* = 2), and preexistent lymphoma (*n* = 1). Transplant from MRD, age younger than 8 years at HSCT and RIC represent the main positive prognostic factor for survival after HSCT in this cohort. Interestingly, not all disease-related conditions responded equally well to transplantation: infections and eczema resolved quicker than food allergies or failure to thrive.

### DOCK2 Deficiency

The Dedicator of cytokinesis 2 (DOCK2) protein is a guanine exchange factor that regulates activation of Rac1 and actin polymerization upon activation of various surface receptors expressed by hematopoietic cells. Dobbs et al. identified biallelic deleterious DOCK2 mutations in 5 unrelated children with early-onset severe bacterial and viral infections, whose immunological phenotype included T cell lymphopenia, markedly reduced number of naïve T cell, defective antibody responses and impaired NK cell function; two of them had reduced B cell count (73). Two of these patients died of viral pneumonitis and of Klebsiella pneumoniae sepsis at 6 years and at 20 months of life, respectively. The remaining three patients were successfully treated by allogeneic HSCT. Different donor types and conditioning regimens were used in these three patients. In particular, one of them received a T-cell-depleted haploidentical transplantation from his father following MAC with busulfan and fludarabine at 9 months of age; a second patient was treated at 3.8 years of life with a MUD HSCT following RIC with treosulfan, fludarabine, and alemtuzumab; a third patient received at 3 years of age MRD HSCT following MAC with busulfan and cyclophosphamide. All three patients have successfully engrafted and are immune reconstituted.

### Functional T Cell Immunodeficiencies

Numerous genetic defects have been identified that allow T cell development but affect proximal or distal steps in intracellular signaling, thereby causing T cell dysfunction (74).

The CD3 complex consists of CD3γ, CD3δ, CD3ε, and CD3ζ chains that associate with the T cell receptor (TCR) and mediate signal transduction. Genetic defects that cause complete **lack of CD3δ or CD3ε** chain expression lead to a block of T cell development and are a cause of SCID (75, 76). **CD3ζ deficiency** is characterized by a reduced number of circulating T cells that are non-functional and display a restricted TCR repertoire (77). Altogether, CD3δ, CD3ε and CD3ζ deficiencies are responsible for a severe immune deficiency and require HSCT early in life (78). However, somatic mosaicism with gene reversion or second-site mutations restoring protein expression may allow residual T cell development and more prolonged survival, leading to atypical presentations of the disease (79). Of note, haploidentical HSCT in the absence of conditioning is typically associated with poor outcome (78, 80). As compared to CD3δ, CD3ε, and CD3ζ deficiencies, mutations in *CD3G* gene causing **CD3γ deficiency** are responsible for a less severe phenotype, with residual production of T cells. These patients are at higher risk of autoimmune manifestations that may occasionally be severe enough to warrant HSCT (81). Ozgür et al. have reported on an infant with CD3γ deficiency and severe inflammatory bowel disease, recurrent pulmonary infections, and candidiasis who received HSCT from his matched sister following RIC with fludarabine, cyclophosphamide and rabbit ATG, but failed to engraft. A second transplant was performed 5 months later from the same donor using a more intense conditioning with busulfan (8 mg/kg), fludarabine and cyclophosphamide. In spite of engraftment and initial signs of immune reconstitution, the patient died of a sudden respiratory distress on day +50 (82). Another patient was reported by Rowe et al. who received MUD HSCT at 32 months of age because of intractable enteropathy, autoimmune hemolytic anemia, and granulomatous-lymphocytic interstitial lung disease (GLILD), but died of severe GVHD at month +15 (81).

The Zeta-chain-associated protein kinase 70 (ZAP-70) was first described in 1994 as a critical molecule for T cell receptor signal transduction (83–85). When the TCR is engaged, ZAP70 is recruited to the plasma membrane where it binds to the phosphorylated CD3ζ. ZAP-70 becomes activated and phosphorylates a number of signal transduction proteins (SLP76, Cbl, Vav) activating several TCR-mediated pathways. Homozygous mutations in the kinase domain of ZAP-70 result in a combined immunodeficiency disease characterized by susceptibility to bacterial, herpes virus, and fungal infections. There is a marked decrease in CD8+ T cells with normal numbers of CD4+ T cells, NK cells, and B cells. In some cases, **ZAP-70 deficiency** may also manifest with atypical clinical and immunological phenotypes, including autoimmunity and immune dysregulation (86–89). The role of HSCT and the optimal donor source and conditioning regimen for ZAP-70 are evolving. Fagioli et al described successful cord blood transplant in two patients using a myeloablative regimen with busulfan, cyclophosphamide, and ATG (90). There was reversal of the phenotype in both patients despite mixed chimerism in the first patient. Kim et al. described emergency transplant in a 9-month-old, critically ill infant with ZAP-70 deficiency using a matched sibling donor, and an infusion of bone marrow cells without conditioning. There was initial engraftment followed by progressive loss of chimerism over the ensuing 3 months (91). However, the initial infusion of stem cells stabilized the clinical situation and allowed a subsequent transplant using the same donor and a myeloablative regimen with busulfan and cyclophosphamide. The largest HSCT study comprising 8 patients was reported by Cuvelier et al. (92). All 8 patients survived and were disease-free. Three matched sibling recipients were transplanted without conditioning and all three had stable, mixed donor T-cell chimerism, but low B-cell chimerism (4–9%) and no or very low myeloid chimerism. Despite the low B-cell chimerism, all three had normal IgG levels and response to vaccinations, and did not require IVIG. Five patients received a myeloablative regimen—three T-cell depleted haplos and two umbilical cord blood-. All five had full donor chimerism in all compartments. Of note, the patients were all very young with ages ranging from 3 weeks to 27 months. In conclusion, HSCT with a chemotherapy-based regimen resulted effective in reversing the clinical and immunological phenotype of ZAP-70 deficiency.

The Linker for Activation of T cells (LAT) is also part of TCR/CD3 signalosome. ***LAT* mutations** in humans have been associated either with a T– B+ NK+ SCID phenotype in infancy (93) or with CID and profound immune dysregulation (manifesting as autoimmune cytopenias and lymphoproliferative disease) allowing survival into late childhood (94). In this latter group of patients, lymphopenia was progressive, and a high proportion of TCRγδ+ T cells was present. Bacchelli et al. have reported on the outcome of HSCT in 5 patients with SCID due to LAT deficiency from a single family (93). Two patients received MRD transplantation without conditioning: one of them was reported to be alive and well, but the other one died of severe acute GVHD. Two patients received MUD HSCT; conditioning was with busulfan and fludarabine, and again one survived and one died of multiorgan failure (MOF) related to toxicity of chemotherapy. Finally, a fifth patient received two haploidentical transplants from the same donor: graft failure was observed after the first unconditioned transplant; the second transplant was performed with conditioning based on melphalan only, but the patient died of MOF. Among the 3 patients with LAT deficiency presenting with CID and immune dysregulation and reported by Keller et al. two died of infections prior to receiving HSCT, whereas the remaining patient was successfully reconstituted after HSCT from his matched sibling donor, who was heterozygous for the LAT mutation (94).

Coronin-1A is involved in neutrophil- and macrophage-mediated phagocytosis and in TCR-induced activation of T lymphocytes. Shiow et al. described a female patient with **bi-allelic loss-of-function mutations in the *CORO1A* gene**, who presented with failure to grow and delayed language and motor development, recurrent upper and lower respiratory tract infections, oral thrush, severe vaccine-associated varicella and rotavirus diarrhea (95). Immunological investigations revealed severe T cell lymphopenia, markedly reduced number of naive CD4+ T cells, poor proliferative response to mitogens and antigens, and impaired antibody response upon immunization with the neoantigen ϕX-174. Unrelated cord blood transplantation was attempted at age 4 years with a RIC regimen consistent of melphalan, fludarabine and alemtuzumab, leading to full donor engraftment, immune reconstitution and resolution of infections. While null mutations in the *CORO1A* gene cause a SCID phenotype, hypomorphic mutations in the same gene are associated with milder clinical and immunological phenotypes, that include T cell lymphopenia, oligoclonal expansion of memory T cells and aggressive EBV-driven lymphoproliferative disease in three siblings (96). One of these patients received haploidentical HSCT from her pheno-identical mother, but died of GVHD.

Recent data indicate the need to consider HSCT in the treatment also of other functional T cell defects, even when they present with a delayed-onset. These conditions include Ras Homolog gene family member H (**RhoH) deficiency** (97), T lymphocyte-specific protein tyrosine kinase **(LCK) deficiency** (98, 99), Serine/threonine kinase 4 **(STK4) deficiency** (100, 101), and IL-2-inducible T cell kinase **(ITK) deficiency** (102–106).

### Wiskott-Aldrich Syndrome and Other Immunodeficiencies With Congenital Thrombocytopenia

The **Wiskott-Aldrich syndrome (WAS)** is an X-linked disorder characterized by the triad of immunodeficiency, eczema, and thrombocytopenia. The immunodeficiency may manifest as recurrent and/or chronic infections, autoimmunity and increased susceptibility to malignancies, especially EBV-associated lymphoma (107). WAS has an estimated incidence of 1 in 100,000 live male births, and is caused by mutations of the *WAS* gene, which encodes the WAS protein (WASP), expressed in all non-erythroid hematopoietic cells and involved in signal transduction and cytoskeleton remodeling. The WASP protein plays a crucial role in formation of the immunological synapse and in migration of lymphoid and myeloid cells in response to chemotactic signals (108). Variable severity of the clinical phenotype has been reported in patients with *WAS* mutations. In most cases, patients suffer since early in life from severe eczema, bloody diarrhea, and recurrent infections (107). Otitis media, and bacterial upper and lower respiratory tract infections are particular common, however patients are also at risk for recurrent, chronic and/or severe viral infections, especially those sustained by herpesviruses, papillomavirus, and molluscum contagiosum. Autoimmune manifestations are also frequent, in particular haemolytic anemia, arthritis, inflammatory bowel disease, and IgA nephropathy. There is an increased risk of EBV-driven lymphoproliferative disease, lymphoma and leukemia.

However, in some patients, clinical manifestations of the disease are less severe. In particular, Villa et al. have demonstrated that hypomorphic mutations, and especially missense mutations in exons 2 and 3 of the gene affecting interaction of WASP with WIP and stability of the WASP protein, cause isolated X-linked thrombocytopenia (XLT) (109), which may even be intermittent (110). The immunological phenotype includes progressive lymphopenia, impaired T cell proliferation in response to anti-CD3, defective NK cytolytic function, reduced levels of IgM with elevated IgA and IgE, impaired production of antibodies (especially to polysaccharide antigens), and reduced number of switched memory B cells (107).

Historical data had indicated that in the absence of definitive therapy, WAS males have a short life span (6.5 years) (111), however use of antimicrobial prophylaxis, prompt treatment of infections and of severe autoimmune manifestations, and regular administration of immunoglobulins have led to improved survival, so that most WAS patients reach adulthood. Nonetheless, patients with WAS continue to have decreased life expectancy and poor quality of life due to an increased risk of life-threatening infections, autoimmune complications, severe bleeding episodes, and malignancies. Furthermore, although XLT has been considered a milder form of the disease, with superior survival as compared to typical and severe WAS, nonetheless XLT patients are also prone to the same complications of WAS patients (112). Allogeneic HSCT can cure the disease. Successful HSCT was first reported in 1968 (4), and several large retrospective analyses are now available. Moratto et al. reported on a large, multicenter, retrospective study of 194 patients with WAS who received HSCT between 1980 and 2009 (113). Bone marrow was used as the primary source of stem cells in 78.4% of the patients. The 194 patients received a total of 204 transplantations; 10 patients (6 of which had received a mismatched family donor T-cell-depleted transplant) required a second transplantation because of graft failure. The vast majority of the patients (88.1%) received a myeloablative conditioning with busulfan-cyclophosphamide or busulfan-fludarabine. RIC with either treosulfan or melphalan in association with fludarabine was more often used in patients transplanted in more recent years. Overall survival was 84% and was even higher (89.1% 5-year survival) for those who received HSCT since year 2000. HSCT from mismatched family donors or cord blood was associated with worse survival. Among patients who received unmatched related donor HSCT, those who were transplanted at a young age had more favorable outcome as compared to patients transplanted at age >5 years. Patients who went to transplant in better conditions had a lower rate of post-transplant complications. Stable full chimerism was achieved by 73.3% of the patients who survived at least 1 year. Mixed chimerism was associated with an increase rate of incomplete immune reconstitution and of post-transplant autoimmunity. Furthermore, patients who attained <50% donor myeloid chimerism were at higher risk of persistent thrombocytopenia. Recently, Elfeky et al. reported on 100% overall survival in 34 consecutive patients with WAS who underwent a variety of transplantation procedures at a single center. Graft source, patient age, and conditioning did not influence the development of post-transplantation complications (114).

The WASP-interacting protein (WIP) plays a critical role in stabilizing WASP (115), and is involved in formation of a DOCK8-WIP-WASP complex that links the TCR to the actin cytoskeleton (116). In 2012, the first case of an autosomal recessive immunodeficiency due to mutations of the *WIPF1* gene (causing **WIP deficiency**) was reported in an infant with a history of recurrent bacterial and viral infections and thrombocytopenia, associated with T cell lymphopenia, decreased T cell proliferation to mitogens, reduced NK cell function, and elevated serum IgE, resembling WAS (117). Consistent with a role for WIP in stabilizing WASP, both WIP and WASP protein expression were abrogated in patient's lymphocytes. The patient received an unrelated cord blood transplantation with chemotherapy, and full resolution of the disease was observed. Al Mousa et al. have reported on 4 patients with WIP deficiency manifesting with recurrent infections and thrombocytopenia (118). The first patient received MRD HSCT using MAC (busulfan 16 mg/kg, cyclophosphamide 200 mg/kg) with full donor engraftment, and was reported to be alive and well 12 years after transplant. The second patient received a MDR transplantation with the same regimen, but in spite of donor engraftment she died 3 months post-HSCT secondary to CMV pneumonitis. The third and fourth patients received an unrelated cord blood transplantation; MAC was with busulfan (16 mg/kg), cyclophosphamide (200 mg/kg) and rabbit ATG (40 mg/kg), and both were reported to be alive and well 2 and 4 years post-HSCT, respectively. Pfajfer et al. have described a 2-year-old WIP-deficient male with a history of severe CMV respiratory tract infection requiring mechanical ventilation, intermittent bloody diarrhea, profound CD4+ T cell lymphopenia, thrombocytopenia, and hypergammaglobulinemia (119). Because the clinical conditions were deemed to be too severe to attempt HSCT with conditioning, sequential infusions of peripheral lymphocytes and stem cells from his HLA-phenoidentical, CMV-seropositive mother were performed, resulting in clearance of CMV and sustained mixed donor T cell chimerism. The child was then treated with a TCRαβ/CD19-depleted PBSC transplantation from the mother (120); conditioning was with treosulfan (36 g/m^2^) and melphalan (140 mg/m^2^), and repetitive secondary-prophylactic, CMV-specific donor lymphocyte infusions were given, resulting in successful engraftment with a 1-year follow-up.

Recently, several groups have described **ARPC1B deficiency**, an autosomal recessive form of CID associated with immune dysregulation and platelet abnormalities that resemble what observed in Wiskott-Aldrich syndrome (WAS) (121–123). The Actin-Related Protein Complex 1B (ARPC1B) is a key factor for the assembly and maintenance of the ARP2/3 complex that is involved in actin branching. Somech et al. described two ARPC1B-deficient brothers with clinical and laboratory features suggestive of WAS, including skin rash, thrombocytopenia with bloody diarrhea, and recurrent infections (124). Both patients manifested lymphopenia (affecting CD8+ more than CD4+ T cells), a restricted T cell repertoire with presence of clonotypic expansions, and markedly defective T cell proliferation in response to anti-CD3. One of the patients succumbed of veno-occlusive disease following HSCT from MRD, and the other one died at 5 years of age after adenoviral infection leading to multiorgan failure.

Finally, Brigida et al. have reported biallelic ARPC1B mutations in 6 unrelated patients that presented with an early-onset disease characterized by autoimmune manifestations, thrombocytopenia and severe infections (125). In this series, two patients underwent a TCRαβ/CD19-depleted mobilized PBSC transplantation from parental donors; both patients were reported to be alive and well-after transplant.

### Cartilage Hair Hypoplasia

Cartilage hair hypoplasia (CHH) is an autosomal recessive disorder due to mutations of the non-coding *RMRP* gene, whose transcript is involved in ribosomal RNA processing, mitochondrial DNA replication, and regulation of target gene transcription. Patients with CHH present with disproportionate short stature with metaphyseal dysplasia, thin and sparse hair, increased risk of bone marrow failure, Hirschsprung disease, joint hypermotility, immunodeficiency, immune dysregulation, and increased risk of malignancies. The immunodeficiency of CHH is of variable severity: some patients have minimal abnormalities of cellular and/or humoral immunity, whereas others may manifest with significant hypogammaglobulinemia or with profound T cell immunodeficiency resembling SCID. Such patients are at significantly increased risk of serious infections, including life-threatening varicella (126).

Berthet et al. reported on a female with CHH and a SCID phenotype (127). HSCT was performed from her matched sister at 16 months of age following conditioning with busulfan and cyclophosphamide. The post-transplant course was uneventful, and the child attained full and sustained donor chimerism, with reconstitution of cellular and humoral immunity. Skeletal abnormalities remained unchanged.

Guggenheim et al. described three patients with CHH who received HSCT because of severe lymphopenia and T cell immunodeficiency detected shortly after birth (128). One of them was transplanted from a 9/10 MUD, and another patient received a bone marrow transplantation from her haploidentical father upon *in vitro* T-cell depletion with Campath. Both of these patients received conditioning with busulfan (20 mg/kg) and cyclophosphamide (200 mg/kg). The third patient received an unconditioned MRD HSCT. All three patients achieved rapid and sustained immune reconstitution with a follow up of 5, 15, and 20 years.

Finally, Bordon et al. have reviewed the European experience of HSCT in 16 patients with CHH, 13 of which received transplantation in early childhood, whereas the remaining three were transplanted at adolescence age (129). All patients had signs of combined immunodeficiency with (*n* = 5) or without (*n* = 11) autoimmunity or inflammatory manifestations. Two of them were transplanted after developing EBV-related non-Hodgkin lymphoma. Donors included a MRD in 5 cases, a MUD in 7, a haploidentical parent in 3, and a mismatched unrelated donor in 1 patient. Thirteen patients received MAC with busulfan (16–20 mg/kg) and cyclophosphamide (120–200 mg/kg) or fludarabine. Two patients received a fludarabine/melphalan RIC regimen, and one patient received conditioning with treosulfan, cyclophosphamide and alemtuzumab. Ten of the 16 patients (63%) survived. The six who died included all 3 who had received haploidentical HSCT. Four of the 6 deaths were observed during the first year after transplant, and were due to severe infections (in spite of full donor chimerism in 3 of them). Two late deaths were observed, both with incomplete immune reconstitution. Viral and fungal infections were a significant problem after transplant, and were seen in 8 of the 16 patients. Surviving patients showed significant improvement of lymphocyte count and function. Donor chimerism was assessed in 12 patient, and was full in 9; 3 patients had mixed chimerism (70–80% on myeloid cells, 90–94% on T cells). Lung function stabilized or improved in the 7 patients who had pre-existing bronchiectasis.

### Autosomal Dominant Hyper-IgE Syndrome Due to Dominant Negative *STAT3* Mutations (Job's Syndrome)

Dominant negative germline mutations in *STAT3* result in an autosomal dominant Hyper IgE (AD-HIES) syndrome characterized by eczema, skin abscesses, recurrent pneumonias leading to pneumatocoeles, and skeletal and connective tissue abnormalities. The role of HSCT in AD-HIES is still under investigation. Hsu et al. assessed the extent of donor chimerism necessary to reverse the phenotype in two male patients with *STAT3* mutations and somatic mosaicism (130). Despite 33% donor T-cell chimerism, they still developed infections, including chronic mucocutaneous candidiasis. Yanagimachi et al. reported on the follow-up of two patients treated with HSCT (131). In both patients IL-17 production in circulating cells and serum IgE levels returned to normal after HSCT, and the frequency of infections decreased. However, despite 100% donor chimerism, the first patient developed recurrent pulmonary aspergillosis. The second patient had mixed chimerism 10 years following transplant with CD3 49% donor and whole blood 17% donor. This patient developed new pneumatocoeles after HSCT.

Nester et al. also described an unsuccessful HSCT in a patient who received MRD HSCT, but died 6 months after HSCT from pulmonary fibrosis (132). Similarly, Gennery described a 7-year-old girl who had successful engraftment from a 10/10 HLA matched donor, but 4 years after transplant, the IgE levels started rising, and recurrent staphylococcal and pseudomonas infections developed, leading the authors to conclude that HSCT is not efficacious in AD-HIES (133). However, analysis of outcome at a later follow-up in this patient and in another patient who received HSCT at the same institution at the age of 13 years demonstrated improvement in the frequency and severity of infections in both patients (134).

There were two other reports of successful HSCT for AD-HIES. Goussetis et al. described two teenagers, both with high-grade non-Hodgkins lymphoma, who received myeloablative HSCT with busulfan, cyclophosphamide and etoposide (135). Similarly, Patel et al. carried out a T-cell depleted haploidentical related donor HSCT in a 14-year-old girl (136). At 1 month, the donor CD3 chimerism was 3%, requiring a donor lymphocyte infusion. This increased the donor chimerism to 100% by 2 months following transplant.

In conclusion, while HSCT does not correct extra-hematopoietic manifestations of the disease, including skoliosis which may predispose to respiratory tract infections, it seems to have a beneficial effect on the frequency and severity of infections. If performed early in the course of the disease, along with adequate physical therapy, it may represent a valid therapeutic option, especially for patients with matched donors. Its role in patients who lack matched donors has yet to be established.

IgE levels are typically elevated also in patients with Phosphoglucomutase 3 **(PMG3) deficiency**. PMG3 is an enzyme involved in multiple glycosylation pathways and PMG3 mutations are responsible for an autosomal recessive disease characterized by skeletal dysplasia, severe immunodeficiency, often associated with neurodevelopmental delay and tendency to bone marrow failure (137), and in some patients with renal, intestinal, and heart defects. The immunodeficiency may be as severe as SCID; other patients show variable degrees of cellular and humoral immunodeficiency. Stray-Pedersen reported on two patients with PGM3 deficiency who underwent HSCT from a matched cord blood donor, and a matched sibling at 4 months and 6 years of life, respectively (137). No details on conditioning were provided. Both patients were successfully cured. Bernth-Jensen et al. have reported the case of an infant with PGM3 deficiency presenting with a T– B– NK+ SCID phenotype (138). He received conditioning with treosulfan, fludarabine, and ATG, but died of treatment-related multiorgan failure before transfusion of allogeneic hematopoietic stem cells.

### Chronic Granulomatous Disease (CGD)

Chronic granulomatous disease (CGD) is caused by gene mutations that affect the functionality of the nicotinamide adenine dinucleotide phosphate (NADPH) complex, resulting in defective production of microbicidal reactive oxygen species (CGD) (139). The most common form of CGD is inherited as an X-linked trait (XL-CGD) and reflects mutations of the *CYBB* gene encoding for the gp91^phox^ subunit of the NADPH oxidase complex. Autosomal recessive (AR) forms (AR-CGD) are due to mutations of the genes that encode for the p22^phox^, p47^phox^, p67^phox^, and p40^phox^ subunits. The main clinical features of CGD include recurrent bacterial and fungal infections—in particular, infections from catalase-positive organisms as Staphylococcus aureus, Burkholderia cepacia and Aspergillus species- and a high rate of inflammatory complications, such as inflammatory bowel disease; granuloma formation in the liver, lungs, and skin; and inflammation leading to strictures that affect the gastrointestinal and urinary tracts (140, 141). The annual mortality of patients managed conservatively ranges from 2 to 5% (142) and it has been shown that patients treated with HSCT presented a lower rate of infections and lower rates of hospitalization (143, 144). However, CGD harbors the defect in the myeloid lineage and an adequate level of donor myeloid chimerism is fundamental to successfully correct the clinical phenotype. The use of non-MAC regimens for T lymphocyte-depleted MRD HSCT showed an increase need for donor lymphocyte infusions to correct poor donor chimerism with subsequent increased risk for GVHD and an overall survival of 70% (145). Currently, to limit toxicity in patients often undergoing HSCT with concomitant infections, alkylator-based RIC are used. Gungor et al. reported a TRM of 7% (4/56 patients) and 2-year overall survival of 96% with the use of busulfan-based RIC (18). Morillo-Gutierrez et al. described an approach using a treosulfan-based RIC, with a TRM of 8% (6/70 patients) and 2-year overall survival of 90% (146). However, in both studies a limited number of patients lost the graft, proving the difficulties in finding the balance between adequate ablation and conditioning-related toxicity. Donor choice is another important aspect for CGD. Recently, it has been shown that many female carriers of X-linked CGD present autoimmune manifestations, not related to degree of lyonization, and therefore they may not be ideal as HSC donors (147, 148).

### Diseases of Immune Dysregulation

Outcomes for HSCT in immune dysregulation diseases are currently suboptimal. The timing of HSCT and an aggressive control of autoimmunity and hyperinflammation pre-HSCT are critical for a good outcome. Immunosuppressive agents -including targeted therapies-, such as sirolimus for immune dysregulation, polyendocrinopathy, enteropathy X-linked (IPEX) syndrome (149), sirolimus or phosphoinositide 3-kinase (PI3k) inhibitor for *PIK3CD* mutations (150, 151), Janus kinase (JAK) inhibitors in signal transducer and activator of transcription (STAT)1 and *STAT3* gain-of-function (GOF) (152), abatacept in lipopolysaccharide-responsive and beige-like anchor protein (LRBA) deficiency and cytotoxic T-lymphocyte associated protein 4 (CTLA4) haploinsufficiency (153), could be used to control disease before HSCT.

**Immune dysregulation, polyendocrinopathy, enteropathy, X-linked (IPEX)** is a recessive genetic disease that present in infancy with intractable enteropathy, erythroderma, and severe autoimmune manifestations, including type 1 diabetes mellitus, blood cytopenias, autoimmune hepatitis, nephropathy, and myopathy (154). IPEX is due to mutations in the *FOXP3* gene, which encodes for the Forkhead box protein 3, that plays a crucial role in regulatory T (Treg) cell function and hence in immune tolerance. Consistent with this, most patients with IPEX lack CD4+ CD25+ FOXP3+ Treg cells. Other immune abnormalities include elevated serum IgE, eosinophilia, and elevated levels of autoantibodies. If untreated, IPEX is usually fatal in the first years of life. Medical management of IPEX with immunosuppressive agents (tacrolimus, rapamycin, and others) may alleviate symptoms of the disease, but also exposes to an increased risk of infections and is often insufficient to control progression of the disease. Several groups have explored the role of allogeneic HSCT in correcting the IPEX phenotype (155–160). The largest series of HSCT for IPEX has been reported by Barzaghi et al. and included 58 patients (161). The majority of the patients (*n* = 37) received RIC (eg, fludarabine plus non-myeloablative doses of busulfan, treosulfan, or melphalan) or minimal intensity (eg, fludarabine plus low dose radiation or cyclophosphamide) conditioning. A minority of patients received MAC included busulfan plus cyclophosphamide, busulfan of >14 mg/kg or cumulative area under the curve of 80–90 mg h/L (when available) plus fludarabine, or treosulfan. Donor types were as follows: MRD (*n* = 31); MUD (*n* = 21); haploidentical donors (*n* = 5); and other mismatched related donor (*n* = 1). Stem cells sources included bone marrow (*n* = 35), PBSC (*n* = 12), and cord blood (*n* = 13). Alemtuzumab or ATG were used in 49 of the 58 patients. Fifteen patients died (26%), and the estimated overall survival at 15 years was 73.2%. The majority of deaths occurred in the first year after HSCT, and were mainly due to infections. Multivariate analysis showed that type of conditioning, donor type, and age at transplantation did not affect survival. The only variable that had a significant impact on survival was the clinical status of the patients at the time of transplant, which was graded using an organ involvement index. Acute GVHD was reported in 19 patients (grade III or IV in 9) while chronic GVHD was observed in 6 of 52 patient who survived beyond 100 days. Full donor chimerism was observed in 31 of 53 patients who were evaluated. However, only 17 of these 31 patients with full donor chimerism were alive and disease-free; three had died, and 11 had autoimmune manifestations or GVHD. Mixed chimerism was observed in 18 patients, and in 50% of them (*n* = 9) it was associated with disease remission. The Treg cells were 100% of donor origin in 3 of these 9 patients. Five of the 18 patients with mixed chimerism have died, and 4 were reported to be alive with autoimmune manifestations. Overall, a similar remission rate was observed among patients with full (54%) or mixed (50%) chimerism. Graft failure was observed in 4 patients.

*PIK3CD* and *PIK3R1* genes encode for a p110δ catalytic molecule and a p85α regulatory subunit, respectively. These proteins are part of the heterodimeric complex phosphoinositide 3-kinase (PI3K)-δ that catalyzes conversion of phosphatidylinositol (4,5) bisphosphate to phosphatidylinositol (3,4,5) trisphosphate, which represents a second messenger for AKT kinase activation, leading to phosphorylation and inactivation of the transcription factor FOXO1 and activation of the mammalian target of rapamycin (mTOR) and S6 kinase (162). **Type 1 and type 2 activated PI3K-δ syndrome (APDS)** are caused by heterozygous gain-of-function mutations of p110δ and exon-skipping mutations of p85α, respectively, both resulting in hyperactivation of the PI3Kδ complex (163, 164). Clinically, these patients present with immunodeficiency—mainly characterized by acute and chronic viral infections, and recurrent respiratory infections since childhood- and immune dysregulation with autoimmune manifestations, lymphoproliferative disease and increased risk of lymphoma. A variable degree of hypogammaglobulinemia, increased frequency of transitional B cells associated with progressive B-cell lymphopenia and a decrease in naïve T lymphocytes with increased proportion of effector/effector memory T cells represent the most typical immunologic abnormalities (150, 165, 166). Conservative treatment with antibiotic prophylaxis and intravenous immunoglobulins is not always sufficient to manage infections and immunosuppressive agents may not adequately control the development of life-threatening episodes of lymphoproliferation. HSCT represent a potentially curative treatment. Recently, two case series by Nademi et al. (167) and Okano et al. (168) reported on APDS patients treated with HSCT. In the two series a comparable survival rate was observed (9/11 and 7/9, respectively). No severe GVHD was reported. HSCT resulted successful in improving humoral immunity. Favorable factors for effective engraftment were HSCT from MRD, use of more intense conditioning, and inclusion of alemtuzumab. On the other hand, a more aggressive conditioning regimen could explain the higher incidence of viral reactivation observed after HSCT.

**Signal Transducer and Activator of Transcription-1 (*STAT1*) GOF** mutations reduce the dephosphorylation of activated STAT1 in the nucleus leading to increased levels of STAT1. This shifts the balance away from STAT3-mediated induction of IL-17 T-cell generation. Levels of IL-17 and IL-22 are decreased. This results in a combined immunodeficiency with variable clinical phenotype that includes both infections and immune dysregulation. In particular, disease manifestations include recurrent chronic mucocutaneous candidiasis (CMC), bacterial and mycobacterial infections, severe viral infections (especially Herpesviridae infections and progressive multifocal leukoencephalopathy), recurrent and severe autoimmune blood cytopenias, autoimmune hypothyroidism, diabetes mellitus, hepatitis, and intracranial hemorrhages (169–172). Treatment with the JAK1/2 inhibitor ruxolitinib has been shown to be effective in reducing the severity of the disease manifestations (152). Leiding et al. reported on 15 patients with *STAT1* GOF mutations treated with HSCT (173). Although the primary engraftment was 74%, there was a 50% rate of secondary graft failure. Thus, there were 6 primary graft failures and 6 secondary graft failures. Only one of the four patients receiving a RIC regimen (fludarabine, melphalan, alemtuzumab) engrafted, indicating that a higher dose regimen was required for reliable engraftment. Three patients who received a myeloablative regimen had complete donor chimerism. However, the results with the myeloablative regimen were complicated by three early deaths before day 50.

**Signal Transducer and Activator of Transcription-3 (*STAT3*) GOF** mutations cause an autosomal dominant disease characterized by early-onset lymphoproliferation, recurrent infections, autoimmunity and, in some cases, growth retardation (174, 175). Forbes et al have reported 6 patients with *STAT3* GOF mutations who have received treatment with Jakinibs and tocilizumab (176); three patients were reported to be alive and stable, albeit with limited follow-up (range: 3–18 months), whereas the remaining three of these had no or minimal response, and ultimately died, including one patient who received HSCT and succumbed of adenovirus pneumonia and post-transplant hemophagocytic lymphohistiocytosis. Milner et al. have reported on two patients with *STAT3* GOF mutations who have received HSCT for refractory autoimmunity (175). One of them received a 10/10 MUD transplant with conditioning consisting of alemtuzumab, treosulfan (36 g/m^2^) and cyclophosphamide (200 mg/kg) and died at day +138 of severe GVHD and adenovirus infection, whereas the other one received HSCT from a 9/10 unrelated donor with reduced intensity conditioning (fludarabine, melphalan, alemtuzumab) and was reported to be alive and in complete remission with 80% donor chimerism.

Cytotoxic T-Lymphocyte antigen 4 (CTLA4) competes with the co-stimulatory receptor CD28 for its ligands CD80 and CD86 on antigen presenting cells. CTLA4 binds these receptors with a higher affinity than CD28 and removes them from the surface, resulting in reduction in APC-mediated activation of T-cells. Furthermore, CTLA-4 plays an important role in Treg function which are responsible for maintaining self-tolerance and immune homeostasis through suppression of T cell proliferation and differentiation (177, 178). **CTLA4 deficiency** due to heterozygous germline mutations in *CTLA4* leading to haploinsufficiency and impaired CTLA4 dimerization or impaired ligand binding, result in an autosomal dominant immune dysregulation syndrome with immunodeficiency (177, 178). In a series of 133 patients, the penetrance was 60–70% (179). The median age of onset was 11 years. Patients manifested with hypogammaglobulinemia, lymphoproliferation, and autoimmune blood cytopenias, as well as lymphomas. mTOR inhibitors have been used to inhibit the CD28 signaling pathway. Slatter et al. described HSCT in 8 patients with CTLA4 deficiency (180). All received MUD after reduced intensity conditioning. Six of the 8 patients are alive and well with donor chimerisms of 85–100%. Two patients received only fludarabine and 4 Gy TBI and both did well. Of note, four of the 8 patients developed GVHD despite having well-matched donors and three of the four received alemtuzumab, suggesting a role for pre-transplant immunosuppression or post-transplant cyclophosphamide.

Mutations in LPS-responsive, beige-like anchor (*LRBA*) are responsible for an immune dysregulation syndrome resulting in a heterogeneous phenotype characterized by hypogammaglobulinemia with B-cell deficiency, functional T-cell defects, aberrant autophagy, autoimmunity and chronic diarrhea (181). LRBA acts as a chaperone for CTLA4, enabling recycling of the molecule and the suppressive capacity of T regs. In the absence of LRBA, CTLA4 is targeted to lysosomal degradation (153). HSCT has been carried out in **LRBA deficiency**. Bakhtiar et al. described a 12 year old with enteropathy, polyarthritis, and immune hemolytic anemia who received a matched sibling transplant from his 15 year-old brother after conditioning with melphalan 140 mg/m^2^, thiotepa 10 mg/kg, fludarabine, and ATG 20 mg/kg/day for 3 days (182). The patient was 100% donor by day 19 post-HSCT, and this level of chimerism remained stable, leading to reversal of the disease phenotype. Tesi et al. reported on a 15 year old male with LRBA deficiency with immune hemolytic anemia, low IgG, low B-cells, and low NK cells (183). HSCT from her HLA pheno-identical mother after conditioning with Treosulfan 14 g/m^2^/day ×3 days, fludarabine, and ATG resulted in 98% donor chimerism and reversal of the phenotype. Seidel et al described a 10-year-old who received an HSCT from her HLA matched mother after conditioning with melphalan, ATG, and fludarabine. Chimerism was 100% donor (184). Finally, Gamez-Diaz et al. have reported on three patients who received HSCT for LRBA deficiency (185). Two of them were successfully cured, after receiving HSCT at the age of 10 and 12 years from the HLA-phenoidentical mother and an HLA-matched sibling, respectively. Conditioning in both was with fludarabine (150 and 160 mg/m^2^, respectively), melphalan (140 mg/m^2^) and ATG (60 mg/kg); thiotepa (10 mg/kg) was added in the conditioning regimen for the second patient. Bone marrow was used as source of stem cells. The first patient developed adenovirus viremia post-HSCT. Other complications in this patient included suspected graft failure with pancytopenia at day +60 requiring a stem cell boost, and hepatitis, immune thrombocytopenia and vitiligo (as signs of chronic GVHD) at 3–8 years post-transplant. Both patients are alive at 10 and 5 years after HSCT, respectively. A third patient received HSCT using PBSC from the haploidentical mother at 6 years of age. Conditioning was with fludarabine (160 mg/m^2^), melphalan (140 mg/m^2^), thiotepa (10 mg/kg), and ATG (9 mg/kg). The patient developed adenovirus viremia from day +13 and died of adenovirus pneumonia at day +91.

Two forms of **X-linked lymphoproliferative (XLP)** disease are known. The first disease, also known as **XLP1**, is due to mutations of the *SH2D1A* gene, which encodes the SLAM-associated protein (SAP), an adaptor molecule that regulates signaling through SLAM molecules (186). In the absence of SAP, T and NK cell cytotoxic responses are abolished. In addition, the function of T_FH_ cells is also compromised. The clinical phenotype of XLP1 is characterized by increased susceptibility to life-threatening EBV infection, with fulminant infectious mononucleosis, hemophagocytic lymphohistiocytosis (HLH), and EBV-related lymphoma. Aplastic anemia, severe vasculitis, and dysgammaglobulinemia (with low levels of IgG and elevated levels of IgM) are also possible. Following the case series of XLP1 patients treated with HSCT reported by Gross et al. (187) and Lankester et al. (188), Booth et al. reported on a multicenter study of 91 patients with XLP1 (189), 43 of whom received a total of 46 HSCT with a median age at transplant of 6.2 years. Fourteen patients received transplant from a matched family donor, 28 from an unrelated donor, and 4 from a haploidentical donor. Bone marrow was used as source of stem cells in 24 cases, PBSC in 15, and cord blood in 2. Conditioning regimens were myeloablative (busulfan 12–20 mg/kg; cyclophosphamide 50–200 mg/kg; and TBI 5–12 Gy) in 23 transplants and non-myeloablative (fludarabine 30 mg/kg; melphalan 70–140 mg/kg; busulfan 4–12 mg/kg; or TBI 3–5 Gy) in 23. Serotherapy was added in 14 cases. Type of conditioning had no impact on survival or chimerism. Thirty-five patients attained full donor chimerism, while 3 showed mixed chimerism. Thirty-five patients were alive at a follow-up ranging from 6 weeks to 148 months. Prior HLH was the major risk factor for poor outcome, with decreased survival to 50%. Marsh et al. have reported on the use of RIC in HSCT for XLP1 (190). They studied 16 patients who underwent HSCT between 2006 and 2013 with a regimen consisting of alemtuzumab, fludarabine, and melphalan. Fourteen patients received HSCT from a MRD or MUD, and 2 patients were transplanted from a mismatched unrelated donor. No cases of VOD or pulmonary hemorrhage were observed. Five patients (31%) developed mixed chimerism, and only one of them showed declining chimerism, but returned to full donor chimerism after infusion of stem cell boost and donor lymphocytes. One-year survival was 80%, with long term survival estimated at 71%. Survival was similar for patients with or without a prior history of HLH.

XLP type 2 (**XLP2**) is due to mutations of the X-linked inhibitor of apoptosis (*XIAP*) gene, which regulates survival and inflammatory responses. Patients with XLP2 are at high risk for HLH, but not for EBV-related lymphoma. In addition, inflammatory manifestations, and especially Crohn-like bowel disease, are frequently observed. Other variable clinical manifestations include severe infectious mononucleosis, fistulating skin abscesses, celiac-like disease, splenomegaly, and antibody deficiency contributing to recurrent infections (191). In 2013, Marsh et al. reviewed the experience in 19 patients with XLP2 who had received HSCT between 2001 and 2011 at various centers (192). Seven patients received a myeloablative regimen, which in five cases consisted of busulfan, cyclophosphamide, and ATG, with or without etoposide; two patients received busulfan and either fludarabine or melphalan, and ATG. Eleven patients received a RIC regimen, which in 10 cases consisted of fludarabine, melphalan, and alemtuzumab; one patient received fludarabine, treosulfan, thiotepa, and alemtuzumab. The remaining patient received an intermediate protocol consisting of TBI (6Gy), fludarabine, cyclophosphamide, and melphalan. In eleven patients, transplants were performed with MRD (*n* = 2) or MUD (*n* = 9); eight patients received a single antigen-mismatched graft. The stem cell source was represented by bone marrow in 11 patients, cord blood in 5, and PBSC in 3. All patients engrafted, except one who died on day +13. A high incidence of treatment-related toxicities was observed among recipients of myeloablative conditioning, including 3 cases of fatal VOD. Two of these patients had also developed pulmonary hemorrhages. Six patients developed mixed chimerism; all of these had received a RIC regimen. One of these patients eventually lost the graft, and 3 of the others received a stem cell boost and/or donor lymphocyte infusions, leading to improved (>90%) donor chimerism. Only one of the 7 patients who received a myeloablative regimen was reported to be alive at the time of last follow-up; of the 11 patients who had received a RIC regimen, 6 were reported to be alive. The 1-year probability of survival was 15 and 57% for patients treated with myeloablative and RIC regimens, respectively. Worth et al. reported on a 12-year-old child with XLP2 and refractory HLH who was successfully transplanted with PBSC from a matched unrelated donor upon conditioning with anti-CD45 monoclonal antibody, fludarabine, cyclophosphamide, and alemtuzumab (193). The post-transplant course was complicated by capillary leak syndrome and pulmonary edema requiring ventilation. Because of mixed chimerism and of possible relapsing HLH, he received etoposide, and eventually attained full donor chimerism and disease correction. Tsuma et al. reported on a child with intractable colitis associated with XIAP deficiency who was successfully treated with HSCT from a matched unrelated donor upon conditioning with fludarabine (120 mg/m^2^), melphalan (140 mg/m^2^), total lymphoid irradiation (3Gy), and ATG (2.5 mg/kg) (194). Occurrence of HLH on day +21 required treatment with etoposide and dexamethasone. At 11 months post-transplant, the patient was reported to be alive with full donor chimerism, but presence of hepatic chronic GVHD. Finally, Chellapendian et al. have reported on an infant with HLH due to XIAP deficiency who received HSCT from MUD while on remission (195). Conditioning was with fludarabine (150 mg/m^2^), melphalan (140 mg/m^2^), and alemtuzumab (1 mg/kg). Post-transplant complications included EBV and HHV6 reactivation, and hypertension. Chimerism dropped from 90 to 50% by day +48. The patient received donor lymphocyte infusions, but declining mixed chimerism (down to 10%) continued to be seen, until a T-cell-depleted stem cell boost was performed on day +441, which resulted in increased donor chimerism to 21.5%. The patient was reported to be alive and well at last follow-up.

## Conclusions

HSCT has the potential of providing definitive correction for most PID. The broad spectrum of clinical and immunological phenotypes associated with these diseases makes it difficult to define a universal transplant regimen. Moreover, patients with PID are complex and specific knowledge is required for the correct management both pre- and post-HSCT. As shown reporting on the different approaches to HSCT for individual PID, the decisions regarding the indication and time to transplant must carefully consider the risks of HSCT against the risks of further disease evolution and must be individualized not only on the basis of the specific PID but also on the characteristics of the single patient. The recent advances in HSCT, including reduced toxicity conditioning agents and graft manipulation, have drastically improved the outcome of HSCT in PID. Early identification of infant affected by SCID, prior to infectious complication, through newborn screening programs and prompt genetic diagnosis with Next Generation Sequencing techniques, will also ameliorate HSCT outcome. However, more evidence is required, especially regarding the newly described PID, including disorders associated with immune dysregulation. Integration of knowledge between immunologists and transplant specialists is necessary for the development of innovative transplant protocols and to monitor their results during follow-up.

## Author Contributions

RC analyzed evidence from literature and wrote the manuscript. OD analyzed evidence from literature and helped supervise the project. EC elaborated the tables. LN supervised the project and critically reviewed the final draft. All the authors approved the submitted manuscript and agreed to be accountable for the content of the work.

### Conflict of Interest Statement

The authors declare that the research was conducted in the absence of any commercial or financial relationships that could be construed as a potential conflict of interest.

## References

[1] Picard C Bobby Gaspar H Al-Herz W Bousfiha A Casanova J-L Chatila T. International Union of Immunological Societies: 2017 Primary Immunodeficiency Diseases Committee Report on Inborn Errors of Immunity. J Clin Immunol. (2018) 38:96–128. 10.1007/s10875-017-0464-9 29226302 PMC5742601

[2] Bousfiha A Jeddane L Picard C Ailal F Bobby Gaspar H Al-Herz W. The 2017 IUIS phenotypic classification for primary immunodeficiencies. J Clin Immunol. (2018) 38:129–43. 10.1007/s10875-017-0465-8 29226301 PMC5742599

[3] Bach FH Amos DB. Hu-1: major histocompatibility locus in man. Science. (1967) 156:1506–8. 10.1126/science.156.3781.1506 4887739

[4] Bach F Albertini R Joo P Anderson J Bortin M Bone-marrow transplantation in a patient with the Wiskott-Aldrich syndrome. Lancet. (1968) 292:1364–6. 10.1016/S0140-6736(68)92672-X 4177931

[5] Gatti R Meuwissen H Allen H Hong R Good R Immunological reconstitution of sex-linked lymphopenic immunological deficiency. Lancet. (1968) 292:1366–9. 10.1016/S0140-6736(68)92673-1 4177932

[6] Laberko A Gennery AR. Clinical considerations in the hematopoietic stem cell transplant management of primary immunodeficiencies. Expert Rev Clin Immunol. (2018) 14:297–306. 10.1080/1744666X.2018.1459189 29589971

[7] Slatter MA Gennery AR. Hematopoietic cell transplantation in primary immunodeficiency – conventional and emerging indications. Expert Rev Clin Immunol. (2018) 14:103–14. 10.1080/1744666X.2018.1424627 29300535

[8] Fox TA Chakraverty R Burns S Carpenter B Thomson K Lowe D. Successful outcome following allogeneic hematopoietic stem cell transplantation in adults with primary immunodeficiency. Blood. (2018) 131:917–31. 10.1182/blood-2017-09-807487 29279357 PMC6225386

[9] Al-Herz W Chou J Delmonte OM Massaad MJ Bainter W Castagnoli R. Comprehensive genetic results for primary immunodeficiency disorders in a highly consanguineous population. Front Immunol. (2019) 9:3146. 10.3389/fimmu.2018.03146 30697212 PMC6340972

[10] Pai S-Y Logan BR Griffith LM Buckley RH Parrott RE Dvorak CC. Transplantation outcomes for severe combined immunodeficiency, 2000–2009. N Engl J Med. (2014) 371:434–46. 10.1056/NEJMoa1401177 25075835 PMC4183064

[11] Bertaina A Merli P Rutella S Pagliara D Bernardo ME Masetti R. HLA-haploidentical stem cell transplantation after removal of α*β*+ T and B cells in children with nonmalignant disorders. Blood. (2014) 124:822–6. 10.1182/blood-2014-03-563817 24869942

[12] Balashov D Shcherbina A Maschan M Trakhtman P Skvortsova Y Shelikhova L. Single-center experience of unrelated and haploidentical stem cell transplantation with TCRα*β* and CD19 depletion in children with primary immunodeficiency syndromes. Biol Blood Marrow Transplant. (2015) 21:1955–62. 10.1016/j.bbmt.2015.07.008 26187864

[13] Shah RM Elfeky R Nademi Z Qasim W Amrolia P Chiesa R. T-cell receptor α*β*+ and CD19+ cell–depleted haploidentical and mismatched hematopoietic stem cell transplantation in primary immune deficiency. J Allergy Clin Immunol. (2018) 141:1417–1426.e1. 10.1016/j.jaci.2017.07.008 28780238

[14] Elfeky R Shah RM Unni MNM Ottaviano G Rao K Chiesa R. New graft manipulation strategies improve the outcome of mismatched stem cell transplantation in children with primary immunodeficiencies. J Allergy Clin Immunol. (2019) 144:280–93. 10.1016/j.jaci.2019.01.030 30731121

[15] Merli P Bertaina V Galaverna F Algeri M Sinibaldi M Strocchio L Donor T cells genetically modified with a novel suicide gene (inducible Caspase 9, iC9) expand and persist over time after post-allograft infusion in patients given ab T-cell and B-cell depleted HLA-haploidentical allogeneic stem cell transplantation. Blood. (2017) 130(Suppl. 1), 211 Available online at: http://www.bloodjournal.org/content/130/Suppl_1/211/tab-article-info

[16] Touzot F Neven B Dal-Cortivo L Gabrion A Moshous D Cros G. CD45RA depletion in HLA-mismatched allogeneic hematopoietic stem cell transplantation for primary combined immunodeficiency: a preliminary study. J Allergy Clin Immunol. (2015) 135:1303–9.e1-3. 10.1016/j.jaci.2014.08.019 25282016

[17] EBMT/ESID Guidelines for Haematopoietic Stem Cell Transplantation for Primary Immunodeficiencies Available online at: https://esid.org/layout/set/print/Working-Parties/Inborn-Errors-Working-Party-IEWP/Resources/UPDATED!-EBMT-ESID-GUIDELINES-FOR-HAEMATOPOIETIC-STEM-CELL-TRANSPLANTATION-FOR-PI (accessed April 30, 2019).

[18] Güngör T Teira P Slatter M Stussi G Stepensky P Moshous D. Reduced-intensity conditioning and HLA-matched haemopoietic stem-cell transplantation in patients with chronic granulomatous disease: a prospective multicentre study. Lancet. (2014) 383:436–48. 10.1016/S0140-6736(13)62069-3 24161820

[19] Slatter MA Rao K Amrolia P Flood T Abinun M Hambleton S. Treosulfan-based conditioning regimens for hematopoietic stem cell transplantation in children with primary immunodeficiency: United Kingdom experience. Blood. (2011) 117:4367–75. 10.1182/blood-2010-10-312082 21325599

[20] Rao K Adams S Qasim W Allwood Z Worth A Silva J. Effect of stem cell source on long-term chimerism and event-free survival in children with primary immunodeficiency disorders after fludarabine and melphalan conditioning regimen. J Allergy Clin Immunol. (2016) 138:1152–60. 10.1016/j.jaci.2016.01.053 27241891

[21] Kröger N Solano C Wolschke C Bandini G Patriarca F Pini M. Antilymphocyte globulin for prevention of chronic graft-versus-host disease. N Engl J Med. (2016) 374:43–53. 10.1056/NEJMoa1506002 26735993

[22] Shah AJ Kapoor N Crooks GM Weinberg KI Azim HA Killen R. The effects of campath 1H upon graft-versus-host disease, infection, relapse, and immune reconstitution in recipients of pediatric unrelated transplants. Biol Blood Marrow Transplant. (2007) 13:584–93. 10.1016/j.bbmt.2007.01.076 17448918

[23] Klein OR Chen AR Gamper C Loeb D Zambidis E Llosa N. Alternative-donor hematopoietic stem cell transplantation with post-transplantation cyclophosphamide for nonmalignant disorders. Biol Blood Marrow Transplant. (2016) 22:895–901. 10.1016/j.bbmt.2016.02.001 26860634 PMC4898048

[24] Neven B Diana J-S Castelle M Magnani A Rosain J Touzot F. Haploidentical hematopoietic stem cell transplantation with post-transplant cyclophosphamide for primary immunodeficiencies and inherited disorders in children. Biol Blood Marrow Transplant. (2019) 25:1363–73. 10.1016/j.bbmt.2019.03.009 30876929

[25] Zeiser R Blazar BR. Acute graft-versus-host disease — biologic process, prevention, and therapy. N Engl J Med. (2017) 377:2167–79. 10.1056/NEJMra1609337 29171820 PMC6034180

[26] Naik S Nicholas SK Martinez CA Leen AM Hanley PJ Gottschalk SM. Adoptive immunotherapy for primary immunodeficiency disorders with virus-specific T lymphocytes. J Allergy Clin Immunol. (2016) 137:1498–505.e1. 10.1016/j.jaci.2015.12.1311 26920464 PMC4860050

[27] Buckley RH Schiff SE Schiff RI Markert L Williams LW Roberts JL. Hematopoietic stem-cell transplantation for the treatment of severe combined immunodeficiency. N Engl J Med. (1999) 340:508–16. 10.1056/NEJM199902183400703 10021471

[28] Recher M Berglund LJ Avery DT Cowan MJ Gennery AR Smart J. IL-21 is the primary common γ chain-binding cytokine required for human B-cell differentiation *in vivo*. Blood. (2011) 118:6824–35. 10.1182/blood-2011-06-362533 22039266 PMC3338166

[29] Miggelbrink AM Logan BR Buckley RH Parrott RE Dvorak CC Kapoor N. B-cell differentiation and IL-21 response in IL2RG/JAK3 SCID patients after hematopoietic stem cell transplantation. Blood. (2018) 131:2967–77. 10.1182/blood-2017-10-809822 29728406 PMC6024640

[30] Abd Hamid IJ Slatter MA McKendrick F Pearce MS Gennery AR. Long-term outcome of hematopoietic stem cell transplantation for IL2RG/JAK3 SCID: a cohort report. Blood. (2017) 129:2198–201. 10.1182/blood-2016-11-748616 28209722

[31] Puel A Ziegler SF Buckley RH Leonard WJ. Defective IL7R expression in T(-)B(+)NK(+) severe combined immunodeficiency. Nat Genet. (1998) 20:394–7. 9843216 10.1038/3877

[32] Dvorak CC Patel K Puck JM Wahlstrom J Dorsey MJ Adams R. Unconditioned unrelated donor bone marrow transplantation for IL7Rα- and Artemis-deficient SCID. Bone Marrow Transplant. (2017) 52:1036–8. 10.1038/bmt.2017.74 28436970 PMC5774618

[33] Notarangelo LD Kim M-S Walter JE Lee YN. Human RAG mutations: biochemistry and clinical implications. Nat Rev Immunol. (2016) 16:234–46. 10.1038/nri.2016.28 26996199 PMC5757527

[34] Walter JE Rosen LB Csomos K Rosenberg JM Mathew D Keszei M. Broad-spectrum antibodies against self-antigens and cytokines in RAG deficiency. J Clin Invest. (2015) 125:4135–48. 10.1172/JCI80477 26457731 PMC4639965

[35] Delmonte OM Schuetz C Notarangelo LD. RAG Deficiency: two genes, many diseases. J Clin Immunol. (2018) 38:646–55. 10.1007/s10875-018-0537-4 30046960 PMC6643099

[36] Farmer JR Foldvari Z Ujhazi B De Ravin SS Chen K Bleesing JJH. Outcomes and treatment strategies for autoimmunity and hyperinflammation in patients with RAG deficiency. J Allergy Clin Immunol Pract. (2019) 7:1970–85.e4. 10.1016/j.jaip.2019.02.038 30877075 PMC6612449

[37] Schuetz C Neven B Dvorak CC Leroy S Ege MJ Pannicke U. SCID patients with ARTEMIS vs RAG deficiencies following HCT: increased risk of late toxicity in ARTEMIS-deficient SCID. Blood. (2014) 123:281–9. 10.1182/blood-2013-01-476432 24144642 PMC3953035

[38] Haddad E Logan BR Griffith LM Buckley RH Parrott RE Prockop SE. SCID genotype and 6-month posttransplant CD4 count predict survival and immune recovery. Blood. (2018) 132:1737–49. 10.1182/blood-2018-03-840702 30154114 PMC6202916

[39] Kohn DB Hershfield MS Puck JM Aiuti A Blincoe A Gaspar HB. Consensus approach for the management of severe combined immune deficiency caused by adenosine deaminase deficiency. J Allergy Clin Immunol. (2019) 143:852–63. 10.1016/j.jaci.2018.08.024 30194989 PMC6688493

[40] Hassan A Booth C Brightwell A Allwood Z Veys P Rao K. Outcome of hematopoietic stem cell transplantation for adenosine deaminase-deficient severe combined immunodeficiency. Blood. (2012) 120:3615–24. 10.1182/blood-2011-12-396879 22791287

[41] Hoenig M Lagresle-Peyrou C Pannicke U Notarangelo LD Porta F Gennery AR. Reticular dysgenesis: international survey on clinical presentation, transplantation, and outcome. Blood. (2017) 129:2928–38. 10.1182/blood-2016-11-745638 28331055 PMC5445572

[42] Dvorak CC Cowan MJ. Radiosensitive severe combined immunodeficiency disease. Immunol Allergy Clin North Am. (2010) 30:125–42. 10.1016/j.iac.2009.10.004 20113890 PMC2818388

[43] Slack J Albert MH Balashov D Belohradsky BH Bertaina A Bleesing J. Outcome of hematopoietic cell transplantation for DNA double-strand break repair disorders. J Allergy Clin Immunol. (2018) 141:322–8.e10. 10.1016/j.jaci.2017.02.036 28392333 PMC5632132

[44] Picard C Fischer A. Hematopoietic stem cell transplantation and other management strategies for MHC class II deficiency. Immunol Allergy Clin North Am. (2010) 30:173–8. 10.1016/j.iac.2010.01.001 20493394

[45] Gennery AR Slatter MA Grandin L Taupin P Cant AJ Veys P. Transplantation of hematopoietic stem cells and long-term survival for primary immunodeficiencies in Europe: entering a new century, do we do better? J Allergy Clin Immunol. (2010) 126:602–10.e1-11. 10.1016/j.jaci.2010.06.015 20673987

[46] Siepermann M Gudowius S Beltz K Strier U Feyen O Troeger A. MHC class II deficiency cured by unrelated mismatched umbilical cord blood transplantation: case report and review of 68 cases in the literature. Pediatr Transplant. (2011) 15:E80–6. 10.1111/j.1399-3046.2010.01292.x 20214747

[47] Small TN Qasim W Friedrich W Chiesa R Bleesing JJ Scurlock A. Alternative donor SCT for the treatment of MHC class II deficiency. Bone Marrow Transplant. (2013) 48:226–32. 10.1038/bmt.2012.140 23000650

[48] Elfeky R Furtado-Silva JM Chiesa R Rao K Lucchini G Amrolia P. Umbilical cord blood transplantation without *in vivo* T-cell depletion for children with MHC class II deficiency. J Allergy Clin Immunol. (2018) 141:2279–82.e2. 10.1016/j.jaci.2017.10.051 29366700

[49] van Kooten C Banchereau J. Functions of CD40 on B cells, dendritic cells and other cells. Curr Opin Immunol. (1997) 9:330–7. 10.1016/S0952-7915(97)80078-7 9203418

[50] Hayward AR Levy J Facchetti F Notarangelo L Ochs HD Etzioni A. Cholangiopathy and tumors of the pancreas, liver, and biliary tree in boys with X-linked immunodeficiency with hyper-IgM. J Immunol. (1997) 158:977–83. 8993019

[51] Thomas C de Saint Basile G Le Deist F Theophile D Benkerrou M Haddad E. Brief report: correction of X-linked hyper-IgM syndrome by allogeneic bone marrow transplantation. N Engl J Med. (1995) 333:426–9. 7542361 10.1056/NEJM199508173330705

[52] Gennery AR Khawaja K Veys P Bredius RGM Notarangelo LD Mazzolari E. Treatment of CD40 ligand deficiency by hematopoietic stem cell transplantation: a survey of the European experience, 1993-2002. Blood. (2004) 103:1152–7. 10.1182/blood-2003-06-2014 14525761

[53] Jacobsohn DA Emerick KM Scholl P Melin-Aldana H O'Gorman M Duerst R. Nonmyeloablative hematopoietic stem cell transplant for X-linked hyper-immunoglobulin m syndrome with cholangiopathy. Pediatrics. (2004) 113:e122–7. 10.1542/peds.113.2.e122 14754981

[54] Mitsui-Sekinaka K Imai K Sato H Tomizawa D Kajiwara M Nagasawa M. Clinical features and hematopoietic stem cell transplantations for CD40 ligand deficiency in Japan. J Allergy Clin Immunol. (2015) 136:1018–24. 10.1016/j.jaci.2015.02.020 25840720

[55] de la Morena MT Leonard D Torgerson TR Cabral-Marques O Slatter M Aghamohammadi A Long-term outcomes of 176 patients with X-linked hyper-IgM syndrome treated with or without hematopoietic cell transplantation. J Allergy Clin Immunol. (2017) 139:1282–92. 10.1016/j.jaci.2016.07.039 27697500 PMC5374029

[56] Ferrua F Galimberti S Courteille V Slatter MA Booth C Moshous D. Hematopoietic stem cell transplantation for CD40 ligand deficiency: results from an EBMT/ESID-IEWP-SCETIDE-PIDTC study. J Allergy Clin Immunol. (2019) 143:2238–53. 10.1016/j.jaci.2018.12.1010 30660643

[57] Ferrari S Giliani S Insalaco A Al-Ghonaium A Soresina AR Loubser M. Mutations of CD40 gene cause an autosomal recessive form of immunodeficiency with hyper IgM. Proc Natl Acad Sci USA. (2001) 98:12614–9. 10.1073/pnas.221456898 11675497 PMC60102

[58] Kutukculer N Aksoylar S Kansoy S Cetingul N Notarangelo LD. Outcome of hematopoietic stem cell transplantation in hyper-IgM syndrome caused by CD40 deficiency. J Pediatr. (2003) 143:141–2. 10.1016/S0022-3476(03)00274-9 12915844

[59] Mazzolari E Lanzi G Forino C Lanfranchi A Aksu G Ozturk C. First report of successful stem cell transplantation in a child with CD40 deficiency. Bone Marrow Transplant. (2007) 40:279–81. 10.1038/sj.bmt.1705713 17502893

[60] Al-Saud B Al-Jomaie M Al-Ghonaium A Al-Ahmari A Al-Mousa H Al-Muhsen S. Haematopoietic stem cell transplant for hyper-IgM syndrome due to CD40 defects: a single-centre experience. Bone Marrow Transplant. (2018) 54:63–7. 10.1038/s41409-018-0219-0 29884852

[61] Zhang Q Davis JC Lamborn IT Freeman AF Jing H Favreau AJ. Combined immunodeficiency associated with *DOCK8* mutations. N Engl J Med. (2009) 361:2046–55. 10.1056/NEJMoa0905506 19776401 PMC2965730

[62] Engelhardt KR McGhee S Winkler S Sassi A Woellner C Lopez-Herrera G. Large deletions and point mutations involving the dedicator of cytokinesis 8 (DOCK8) in the autosomal-recessive form of hyper-IgE syndrome. J Allergy Clin Immunol. (2009) 124:1289–302.e4. 10.1016/j.jaci.2009.10.038 20004785 PMC2818862

[63] Aydin SE Freeman AF Al-Herz W Al-Mousa HA Arnaout RK Aydin RC. Hematopoietic stem cell transplantation as treatment for patients with DOCK8 deficiency. J Allergy Clin Immunol Pract. (2019) 7:848–55. 10.1016/j.jaip.2018.10.035 30391550 PMC6771433

[64] McDonald DR Massaad MJ Johnston A Keles S Chatila T Geha RS. Successful engraftment of donor marrow after allogeneic hematopoietic cell transplantation in autosomal-recessive hyper-IgE syndrome caused by dedicator of cytokinesis 8 deficiency. J Allergy Clin Immunol. (2010) 126:1304–5.e3. 10.1016/j.jaci.2010.07.034 20810158 PMC2998541

[65] Barlogis V Galambrun C Chambost H Lamoureux-Toth S Petit P Stephan J-L. Successful allogeneic hematopoietic stem cell transplantation for DOCK8 deficiency. J Allergy Clin Immunol. (2011) 128:420–2.e2. 10.1016/j.jaci.2011.03.025 21546070

[66] Boztug H Karitnig-Weiß C Ausserer B Renner ED Albert MH Sawalle-Belohradsky J. Clinical and immunological correction of DOCK8 deficiency by allogeneic hematopoietic stem cell transplantation following a reduced toxicity conditioning regimen. Pediatr Hematol Oncol. (2012) 29:585–94. 10.3109/08880018.2012.714844 22897717

[67] Ghosh S Schuster FR Fuchs I Laws H-J Borkhardt A Meisel R. Treosulfan-based conditioning in DOCK8 deficiency: complete lympho-hematopoietic reconstitution with minimal toxicity. Clin Immunol. (2012) 145:259–61. 10.1016/j.clim.2012.10.003 23128504

[68] Cuellar-Rodriguez J Freeman AF Grossman J Su H Parta M Murdock H. Matched related and unrelated donor hematopoietic stem cell transplantation for DOCK8 deficiency. Biol Blood Marrow Transplant. (2015) 21:1037–45. 10.1016/j.bbmt.2015.01.022 25636378 PMC4426076

[69] Freeman AF Shah NN Parta M Su HC Brofferio A Gradus-Pizlo I. Haploidentical related donor hematopoietic stem cell transplantation with post-transplantation cyclophosphamide for DOCK8 deficiency. J Allergy Clin Immunol Pract. (2016) 4:1239–42.e1. 10.1016/j.jaip.2016.06.028 27641484 PMC5605135

[70] Al-Herz W Chu JI van der Spek J Raghupathy R Massaad MJ Keles S. Hematopoietic stem cell transplantation outcomes for 11 patients with dedicator of cytokinesis 8 deficiency. J Allergy Clin Immunol. (2016) 138:852–9.e3. 10.1016/j.jaci.2016.02.022 27130861 PMC5354354

[71] Shah NN Freeman AF Su H Cole K Parta M Moutsopoulos NM. Haploidentical related donor hematopoietic stem cell transplantation for dedicator-of-cytokinesis 8 deficiency using post-transplantation cyclophosphamide. Biol Blood Marrow Transplant. (2017) 23:980–90. 10.1016/j.bbmt.2017.03.016 28288951 PMC5757872

[72] Uygun DFK Uygun V Reisli I Keleş S Özen A Yilmaz M. Hematopoietic stem cell transplantation from unrelated donors in children with DOCK8 deficiency. Pediatr Transplant. (2017) 21:e13015. 10.1111/petr.13015 28664550

[73] Dobbs K Domínguez Conde C Zhang S-Y Parolini S Audry M Chou J. Inherited DOCK2 deficiency in patients with early-onset invasive infections. N Engl J Med. (2015) 372:2409–22. 10.1056/NEJMoa1413462 26083206 PMC4480434

[74] Notarangelo LD. Functional T cell immunodeficiencies (with T cells present). Annu Rev Immunol. (2013) 31:195–225. 10.1146/annurev-immunol-032712-095927 23298211

[75] Dadi HK Simon AJ Roifman CM. Effect of CD3delta deficiency on maturation of alpha/beta and gamma/delta T-cell lineages in severe combined immunodeficiency. N Engl J Med. (2003) 349:1821–8. 10.1056/NEJMoa031178 14602880

[76] Basile G de Saint Geissmann F Flori E Uring-Lambert B Soudais C Cavazzana-Calvo M Severe combined immunodeficiency caused by deficiency in either the δ or the ε subunit of CD3. J Clin Invest. (2004) 114:1512–7. 10.1172/JCI22588 15546002 PMC525745

[77] Rieux-Laucat F Hivroz C Lim A Mateo V Pellier I Selz F. Inherited and somatic CD3zeta mutations in a patient with T-cell deficiency. N Engl J Med. (2006) 354:1913–21. 10.1056/NEJMoa053750 16672702

[78] Marcus N Takada H Law J Cowan MJ Gil J Regueiro JR. Hematopoietic stem cell transplantation for CD3δ deficiency. J Allergy Clin Immunol. (2011) 128:1050–7. 10.1016/j.jaci.2011.05.031 21757226 PMC4490832

[79] Marin A V Jiménez-Reinoso A Briones AC Muñoz-Ruiz M Aydogmus C Pasick LJ. Primary T-cell immunodeficiency with functional revertant somatic mosaicism in CD247. J Allergy Clin Immunol. (2017) 139:347–9.e8. 10.1016/j.jaci.2016.06.020 27555457

[80] Takada H Ishimura M Hara T. Insufficient immune reconstitution after allogeneic cord blood transplantation without chemotherapy conditioning in patients with SCID caused by CD3δ deficiency. Bone Marrow Transplant. (2016) 51:1131–3. 10.1038/bmt.2016.64 26999462

[81] Rowe JH Delmonte OM Keles S Stadinski BD Dobbs AK Henderson LA. Patients with CD3G mutations reveal a role for human CD3γ in Treg diversity and suppressive function. Blood. (2018) 131:2335–44. 10.1182/blood-2018-02-835561 29653965 PMC5969384

[82] Ozgür TT Asal GT Cetinkaya D Orhan D Kiliç SS Usta Y. Hematopoietic stem cell transplantation in a CD3 gamma-deficient infant with inflammatory bowel disease. Pediatr Transplant. (2008) 12:910–3. 10.1111/j.1399-3046.2008.00957.x 18482219

[83] Arpaia E Shahar M Dadi H Cohen A Roifman CM. Defective T cell receptor signaling and CD8+ thymic selection in humans lacking zap-70 kinase. Cell. (1994) 76:947–58. 10.1016/0092-8674(94)90368-9 8124727

[84] Chan AC Kadlecek TA Elder ME Filipovich AH Kuo WL Iwashima M. ZAP-70 deficiency in an autosomal recessive form of severe combined immunodeficiency. Science. (1994) 264:1599–601. 10.1126/science.8202713 8202713

[85] Elder ME Lin D Clever J Chan AC Hope TJ Weiss A. Human severe combined immunodeficiency due to a defect in ZAP-70, a T cell tyrosine kinase. Science. (1994) 264:1596–9. 10.1126/science.8202712 8202712

[86] Picard C Dogniaux S Chemin K Maciorowski Z Lim A Mazerolles F. Hypomorphic mutation of ZAP70 in human results in a late onset immunodeficiency and no autoimmunity. Eur J Immunol. (2009) 39:1966–76. 10.1002/eji.200939385 19548248

[87] Chan AY Punwani D Kadlecek TA Cowan MJ Olson JL Mathes EF. A novel human autoimmune syndrome caused by combined hypomorphic and activating mutations in ZAP-70. J Exp Med. (2016) 213:155–65. 10.1084/jem.20150888 26783323 PMC4749924

[88] Liu Q Wang Y-P Liu Q Zhao Q Chen X-M Xue X-H. Novel compound heterozygous mutations in ZAP70 in a Chinese patient with leaky severe combined immunodeficiency disorder. Immunogenetics. (2017) 69:199–209. 10.1007/s00251-017-0971-0 28124082

[89] Turul T Tezcan I Artac H de Bruin-Versteeg S Barendregt BH Reisli I. Clinical heterogeneity can hamper the diagnosis of patients with ZAP70 deficiency. Eur J Pediatr. (2009) 168:87–93. 10.1007/s00431-008-0718-x 18509675

[90] Fagioli F Biasin E Berger M Nesi F Saroglia EH Miniero R. Successful unrelated cord blood transplantation in two children with severe combined immunodeficiency syndrome. Bone Marrow Transplant. (2003) 31:133–6. 10.1038/sj.bmt.1703800 12621496

[91] Kim VH Murguia L Schechter T Grunebaum E Roifman CM. Emergency treatment for ζ chain-associated protein of 70 kDa (ZAP70) deficiency. J Allergy Clin Immunol. (2013) 131:1233–5. 10.1016/j.jaci.2012.09.020 23141738

[92] Cuvelier GDE Rubin TS Wall DA Schroeder ML. Long-term outcomes of hematopoietic stem cell transplantation for ZAP70 deficiency. J Clin Immunol. (2016) 36:713–24. 10.1007/s10875-016-0316-z 27438785

[93] Bacchelli C Moretti FA Carmo M Adams S Stanescu HC Pearce K. Mutations in linker for activation of T cells (LAT) lead to a novel form of severe combined immunodeficiency. J Allergy Clin Immunol. (2017) 139:634–42.e5. 10.1016/j.jaci.2016.05.036 27522155

[94] Keller B Zaidman I Yousefi OS Hershkovitz D Stein J Unger S. Early onset combined immunodeficiency and autoimmunity in patients with loss-of-function mutation in LAT. J Exp Med. (2016) 213:1185–99. 10.1084/jem.20151110 27242165 PMC4925012

[95] Shiow LR Roadcap DW Paris K Watson SR Grigorova IL Lebet T. The actin regulator coronin 1A is mutant in a thymic egress-deficient mouse strain and in a patient with severe combined immunodeficiency. Nat Immunol. (2008) 9:1307–15. 10.1038/ni.1662 18836449 PMC2672406

[96] Moshous D Martin E Carpentier W Lim A Callebaut I Canioni D. Whole-exome sequencing identifies Coronin-1A deficiency in 3 siblings with immunodeficiency and EBV-associated B-cell lymphoproliferation. J Allergy Clin Immunol. (2013) 131:1594–603. 10.1016/j.jaci.2013.01.042 23522482 PMC3824285

[97] Crequer A Troeger A Patin E Ma CS Picard C Pedergnana V. Human RHOH deficiency causes T cell defects and susceptibility to EV-HPV infections. J Clin Invest. (2012) 122:3239–47. 10.1172/JCI62949 22850876 PMC3428089

[98] Hauck F Randriamampita C Martin E Gerart S Lambert N Lim A. Primary T-cell immunodeficiency with immunodysregulation caused by autosomal recessive LCK deficiency. J Allergy Clin Immunol. (2012) 130:1144–52.e11. 10.1016/j.jaci.2012.07.029 22985903

[99] Li S-L Duo L-N Wang H-J Dai W Zhou E-YH Xu Y-N. Identification of LCK mutation in a family with atypical epidermodysplasia verruciformis with T-cell defects and virus-induced squamous cell carcinoma. Br J Dermatol. (2016) 175:1204–9. 10.1111/bjd.14679 27087313

[100] Abdollahpour H Appaswamy G Kotlarz D Diestelhorst J Beier R Schaffer AA. The phenotype of human STK4 deficiency. Blood. (2012) 119:3450–7. 10.1182/blood-2011-09-378158 22294732 PMC3325036

[101] Lum SH Bonney D Cheesman E Wrignt NB Hughes S Wynn R. Successful curative therapy with rituximab and allogeneic haematopoietic stem cell transplantation for MALT lymphoma associated with STK4-mutated CD4+ lymphocytopenia. Pediatr Blood Cancer. (2016) 63:1657–9. 10.1002/pbc.26048 27163767

[102] Huck K Feyen O Niehues T Rüschendorf F Hübner N Laws H-J. Girls homozygous for an IL-2-inducible T cell kinase mutation that leads to protein deficiency develop fatal EBV-associated lymphoproliferation. J Clin Invest. (2009) 119:1350–8. 10.1172/JCI37901 19425169 PMC2673872

[103] Stepensky P Weintraub M Yanir A Revel-Vilk S Krux F Huck K. IL-2-inducible T-cell kinase deficiency: clinical presentation and therapeutic approach. Haematologica. (2011) 96:472–6. 10.3324/haematol.2010.033910 21109689 PMC3046282

[104] Mansouri D Mahdaviani SA Khalilzadeh S Mohajerani SA Hasanzad M Sadr S. IL-2-inducible T-cell kinase deficiency with pulmonary manifestations due to disseminated Epstein-Barr virus infection. Int Arch Allergy Immunol. (2012) 158:418–22. 10.1159/000333472 22487848

[105] Serwas NK Cagdas D Ban SA Bienemann K Salzer E Tezcan I. Identification of ITK deficiency as a novel genetic cause of idiopathic CD4+ T-cell lymphopenia. Blood. (2014) 124:655–7. 10.1182/blood-2014-03-564930 25061172 PMC4110665

[106] Ghosh S Drexler I Bhatia S Adler H Gennery AR Borkhardt A Interleukin-2-inducible T-cell kinase deficiency—new patients, new insight? Front Immunol. (2018) 9:979 10.3389/fimmu.2018.02197 29867957 PMC5951928

[107] Candotti F. Clinical manifestations and pathophysiological mechanisms of the wiskott-aldrich syndrome. J Clin Immunol. (2018) 38:13–27. 10.1007/s10875-017-0453-z 29086100

[108] Blundell MP Worth A Bouma G Thrasher AJ. The Wiskott-Aldrich syndrome: The actin cytoskeleton and immune cell function. Dis Markers. (2010) 29:157–75. 10.1155/2010/781523 21178275 PMC3835520

[109] Villa A Notarangelo L Macchi P Mantuano E Cavagni G Brugnoni D. X–linked thrombocytopenia and Wiskott–Aldrich syndrome are allelic diseases with mutations in the WASP gene. Nat Genet. (1995) 9:414–7. 10.1038/ng0495-414 7795648

[110] Notarangelo LD Mazza C Giliani S D'Aria C Gandellini F Ravelli C. Missense mutations of the WASP gene cause intermittent X-linked thrombocytopenia. Blood. (2002) 99:2268–9. 10.1182/blood.V99.6.2268 11877312

[111] Sullivan KE Mullen CA Blaese RM Winkelstein JA. A multiinstitutional survey of the Wiskott-Aldrich syndrome. J Pediatr. (1994) 125(Pt 1):876–85. 10.1016/S0022-3476(05)82002-5 7996359

[112] Albert MH Bittner TC Nonoyama S Notarangelo LD Burns S Imai K. X-linked thrombocytopenia (XLT) due to WAS mutations: clinical characteristics, long-term outcome, and treatment options. Blood. (2010) 115:3231–8. 10.1182/blood-2009-09-239087 20173115

[113] Moratto D Giliani S Bonfim C Mazzolari E Fischer A Ochs HD. Long-term outcome and lineage-specific chimerism in 194 patients with Wiskott-Aldrich syndrome treated by hematopoietic cell transplantation in the period 1980-2009: an international collaborative study. Blood. (2011) 118:1675–84. 10.1182/blood-2010-11-319376 21659547 PMC3156052

[114] Elfeky RA Furtado-Silva JM Chiesa R Rao K Amrolia P Lucchini G. One hundred percent survival after transplantation of 34 patients with Wiskott-Aldrich syndrome over 20 years. J Allergy Clin Immunol. (2018) 142:1654–6.e7. 10.1016/j.jaci.2018.06.042 30055182

[115] Sasahara Y. WASP-WIP complex in the molecular pathogenesis of Wiskott-Aldrich syndrome. Pediatr Int. (2016) 58:4–7. 10.1111/ped.12819 26331277

[116] Janssen E Tohme M Hedayat M Leick M Kumari S Ramesh N. A DOCK8-WIP-WASp complex links T cell receptors to the actin cytoskeleton. J Clin Invest. (2016) 126:3837–51. 10.1172/JCI85774 27599296 PMC5096816

[117] Lanzi G Moratto D Vairo D Masneri S Delmonte O Paganini T. A novel primary human immunodeficiency due to deficiency in the WASP-interacting protein WIP. J Exp Med. (2012) 209:29–34. 10.1084/jem.20110896 22231303 PMC3260865

[118] Al-Mousa H Hawwari A Al-Ghonaium A Al-Saud B Al-Dhekri H Al-Muhsen S. Hematopoietic stem cell transplantation corrects WIP deficiency. J Allergy Clin Immunol. (2017) 139:1039–40.e4. 10.1016/j.jaci.2016.08.036 27742395

[119] Pfajfer L Seidel MG Houmadi R Rey-Barroso J Hirschmugl T Salzer E. WIP deficiency severely affects human lymphocyte architecture during migration and synapse assembly. Blood. (2017) 130:1949–53. 10.1182/blood-2017-04-777383 28903942

[120] Schwinger W Urban C Ulreich R Sperl D Karastaneva A Strenger V. The phenotype and treatment of WIP deficiency: literature synopsis and review of a patient with pre-transplant serial donor lymphocyte infusions to eliminate CMV. Front Immunol. (2018) 9:2554. 10.3389/fimmu.2018.02554 30450104 PMC6224452

[121] Kuijpers TW Tool ATJ van der Bijl I de Boer M van Houdt M de Cuyper IM. Combined immunodeficiency with severe inflammation and allergy caused by ARPC1B deficiency. J Allergy Clin Immunol. (2017) 140:273–7.e10. 10.1016/j.jaci.2016.09.061 27965109

[122] Kahr WHA Pluthero FG Elkadri A Warner N Drobac M Chen CH. Loss of the Arp2/3 complex component ARPC1B causes platelet abnormalities and predisposes to inflammatory disease. Nat Commun. (2017) 8:14816. 10.1038/ncomms14816 28368018 PMC5382316

[123] Volpi S Cicalese MP Tuijnenburg P Tool ATJ Cuadrado E Abu-Halaweh M. A combined immunodeficiency with severe infections, inflammation, and allergy caused by ARPC1B deficiency. J Allergy Clin Immunol. (2019) 143:2296–9. 10.1016/j.jaci.2019.02.003 30771411 PMC6677392

[124] Somech R Lev A Lee YN Simon AJ Barel O Schiby G. Disruption of thrombocyte and T lymphocyte development by a mutation in ARPC1B. J Immunol. (2017) 199:4036–45. 10.4049/jimmunol.1700460 29127144 PMC5726601

[125] Brigida I Zoccolillo M Cicalese MP Pfajfer L Barzaghi F Scala S. T-cell defects in patients with ARPC1B germline mutations account for combined immunodeficiency. Blood. (2018) 132:2362–74. 10.1182/blood-2018-07-863431 30254128 PMC6265646

[126] Mäkitie O Vakkilainen S Cartilage-Hair Hypoplasia – Anauxetic Dysplasia Spectrum Disorders. GeneReviews®. Seattle, WA: University of Washington (2018). 22420014

[127] Berthet F Siegrist CA Ozsahin H Tuchschmid P Eich G Superti-Furga A Bone marrow transplantation in cartilage-hair hypoplasia: correction of the immunodeficiency but not of the chondrodysplasia. Eur J Pediatr. (1996) 155:286–90. 10.1007/BF02002714 8777921

[128] Guggenheim R Somech R Grunebaum E Atkinson A Roifman CM. Bone marrow transplantation for cartilage-hair-hypoplasia. Bone Marrow Transplant. (2006) 38:751–6. 10.1038/sj.bmt.1705520 17041608

[129] Bordon V Gennery AR Slatter MA Vandecruys E Laureys G Veys P. Clinical and immunologic outcome of patients with cartilage hair hypoplasia after hematopoietic stem cell transplantation. Blood. (2010) 116:27–35. 10.1182/blood-2010-08-301101 20375313

[130] Hsu AP Sowerwine KJ Lawrence MG Davis J Henderson CJ Zarember KA. Intermediate phenotypes in patients with autosomal dominant hyper-IgE syndrome caused by somatic mosaicism. J Allergy Clin Immunol. (2013) 131:1586–93. 10.1016/j.jaci.2013.02.038 23623265 PMC4103905

[131] Yanagimachi M Ohya T Yokosuka T Kajiwara R Tanaka F Goto H. The potential and limits of hematopoietic stem cell transplantation for the treatment of autosomal dominant hyper-IgE syndrome. J Clin Immunol. (2016) 36:511–6. 10.1007/s10875-016-0278-1 27091139

[132] Nester TA Wagnon AH Reilly WF Spitzer G Kjeldsberg CR Hill HR. Effects of allogeneic peripheral stem cell transplantation in a patient with job syndrome of hyperimmunoglobulinemia E and recurrent infections. Am J Med. (1998) 105:162–4. 10.1016/S0002-9343(98)00200-9 9727824

[133] Gennery A Flood T Abinun M Cant A Bone marrow transplantation does not correct the hyper IgE syndrome. Bone Marrow Transplant. (2000) 25:1303–5. 10.1038/sj.bmt.1702446 10871737

[134] Flinn AM Cant A Leahy TR Butler KM Gennery AR. Autosomal dominant hyper IgE syndrome–treatment strategies and clinical outcomes. J Clin Immunol. (2016) 36:107–9. 10.1007/s10875-015-0231-8 26743515

[135] Goussetis E Peristeri I Kitra V Traeger-Synodinos J Theodosaki M Psarra K. Successful long-term immunologic reconstitution by allogeneic hematopoietic stem cell transplantation cures patients with autosomal dominant hyper-IgE syndrome. J Allergy Clin Immunol. (2010) 126:392–4. 10.1016/j.jaci.2010.05.005 20584545

[136] Patel NC Gallagher JL Torgerson TR Gilman AL. Successful haploidentical donor hematopoietic stem cell transplant and restoration of STAT3 function in an adolescent with autosomal dominant hyper-IgE syndrome. J Clin Immunol. (2015) 35:479–85. 10.1007/s10875-015-0167-z 25962528

[137] Stray-Pedersen A Backe PH Sorte HS Mørkrid L Chokshi NY Erichsen HC. PGM3 mutations cause a congenital disorder of glycosylation with severe immunodeficiency and skeletal dysplasia. Am J Hum Genet. (2014) 95:96–107. 10.1016/j.ajhg.2014.05.007 24931394 PMC4085583

[138] Bernth-Jensen JM Holm M Christiansen M. Neonatal-onset T(-)B(-)NK(+) severe combined immunodeficiency and neutropenia caused by mutated phosphoglucomutase 3. J Allergy Clin Immunol. (2016) 137:321–4. 10.1016/j.jaci.2015.07.047 26409661

[139] Segal BH Leto TL Gallin JI Malech HL Holland SM. Genetic, biochemical, and clinical features of chronic granulomatous disease. Medicine. (2000) 79:170–200. 10.1097/00005792-200005000-00004 10844936

[140] Winkelstein JA Marino MC Johnston RB Boyle J Curnutte J Gallin JI. Chronic granulomatous disease. Report on a national registry of 368 patients. Medicine. (2000) 79:155–69. 10.1097/00005792-200005000-00003 10844935

[141] Rosenzweig SD. Inflammatory manifestations in chronic granulomatous disease (CGD). J Clin Immunol. (2008) 28:67–72. 10.1007/s10875-007-9160-5 18193341

[142] Martire B Rondelli R Soresina A Pignata C Broccoletti T Finocchi A. Clinical features, long-term follow-up and outcome of a large cohort of patients with Chronic granulomatous disease: an Italian multicenter study. Clin Immunol. (2008) 126:155–64. 10.1016/j.clim.2007.09.008 18037347

[143] Cole T Pearce MS Cant AJ Cale CM Goldblatt D Gennery AR Clinical outcome in children with chronic granulomatous disease managed conservatively or with hematopoietic stem cell transplantation. J Allergy Clin Immunol. (2013) 132:1150–5. 10.1016/j.jaci.2013.05.031 23870668

[144] Notarangelo LD. The long road to optimal management for chronic granulomatous disease. J Allergy Clin Immunol. (2013) 132:1164–5. 10.1016/j.jaci.2013.08.035 24094546

[145] Horwitz ME Barrett AJ Brown MR Carter CS Childs R Gallin JI. Treatment of chronic granulomatous disease with nonmyeloablative conditioning and a T-cell-depleted hematopoietic allograft. N Engl J Med. (2001) 344:881–8. 10.1056/NEJM200103223441203 11259721

[146] Morillo-Gutierrez B Beier R Rao K Burroughs L Schulz A Ewins A-M. Treosulfan-based conditioning for allogeneic HSCT in children with chronic granulomatous disease: a multicenter experience. Blood. (2016) 128:440–8. 10.1182/blood-2016-10-745455 27216217 PMC4957165

[147] Battersby AC Braggins H Pearce MS Cale CM Burns SO Hackett S. Inflammatory and autoimmune manifestations in X-linked carriers of chronic granulomatous disease in the United Kingdom. J Allergy Clin Immunol. (2017) 140:628–30.e6. 10.1016/j.jaci.2017.02.029 28343844

[148] Marciano BE Zerbe CS Falcone EL Ding L DeRavin SS Daub J. X-linked carriers of chronic granulomatous disease: Illness, lyonization, and stability. J Allergy Clin Immunol. (2018) 141:365–71. 10.1016/j.jaci.2017.04.035 28528201

[149] Yong PL Russo P Sullivan KE. Use of Sirolimus in IPEX and IPEX-Like Children. J Clin Immunol. (2008) 28:581–7. 10.1007/s10875-008-9196-1 18481161

[150] Maccari ME Abolhassani H Aghamohammadi A Aiuti A Aleinikova O Bangs C. Disease evolution and response to rapamycin in activated phosphoinositide 3-kinase δ syndrome: the European Society for immunodeficiencies-activated phosphoinositide 3-kinase δ syndrome registry. Front Immunol. (2018) 9:543. 10.3389/fimmu.2018.00338 29599784 PMC5863269

[151] Rao VK Webster S Dalm VASH Šedivá A van Hagen PM Holland S. Effective &quot;activated PI3Kδ syndrome&quot;-targeted therapy with the PI3Kδ inhibitor leniolisib. Blood. (2017) 130:2307–16. 10.1182/blood-2017-08-801191 28972011 PMC5701526

[152] Weinacht KG Charbonnier L-M Alroqi F Plant A Qiao Q Wu H. Ruxolitinib reverses dysregulated T helper cell responses and controls autoimmunity caused by a novel signal transducer and activator of transcription 1 (STAT1) gain-of-function mutation. J Allergy Clin Immunol. (2017) 139:1629–40.e2. 10.1016/j.jaci.2016.11.022 28139313 PMC5482293

[153] Lo B Zhang K Lu W Zheng L Zhang Q Kanellopoulou C. Patients with LRBA deficiency show CTLA4 loss and immune dysregulation responsive to abatacept therapy. Science. (2015) 349:436–40. 10.1126/science.aaa1663 26206937

[154] Cepika A-M Sato Y Liu JM-H Uyeda MJ Bacchetta R Roncarolo MG. Tregopathies: monogenic diseases resulting in regulatory T-cell deficiency. J Allergy Clin Immunol. (2018) 142:1679–95. 10.1016/j.jaci.2018.10.026 30527062

[155] Baud O Goulet O Canioni D Le Deist F Radford I Rieu D. Treatment of the immune dysregulation, polyendocrinopathy, enteropathy, X-linked syndrome (IPEX) by allogeneic bone marrow transplantation. N Engl J Med. (2001) 344:1758–62. 10.1056/NEJM200106073442304 11396442

[156] Rao A Kamani N Filipovich A Lee SM Davies SM Dalal J. Successful bone marrow transplantation for IPEX syndrome after reduced-intensity conditioning. Blood. (2007) 109:383–5. 10.1182/blood-2006-05-025072 16990602

[157] Seidel MG Fritsch G Lion T Jurgens B Heitger A Bacchetta R. Selective engraftment of donor CD4+25high FOXP3-positive T cells in IPEX syndrome after nonmyeloablative hematopoietic stem cell transplantation. Blood. (2009) 113:5689–91. 10.1182/blood-2009-02-206359 19478054

[158] Kasow KA Morales-Tirado VM Wichlan D Shurtleff SA Abraham A Persons DA. Therapeutic *in vivo* selection of thymic-derived natural T regulatory cells following non-myeloablative hematopoietic stem cell transplant for IPEX. Clin Immunol. (2011) 141:169–76. 10.1016/j.clim.2011.07.005 21865090 PMC3200481

[159] Burroughs LM Torgerson TR Storb R Carpenter PA Rawlings DJ Sanders J. Stable hematopoietic cell engraftment after low-intensity nonmyeloablative conditioning in patients with immune dysregulation, polyendocrinopathy, enteropathy, X-linked syndrome. J Allergy Clin Immunol. (2010) 126:1000–5. 10.1016/j.jaci.2010.05.021 20643476 PMC2962731

[160] Kucuk ZY Bleesing JJ Marsh R Zhang K Davies S Filipovich AH. A challenging undertaking: stem cell transplantation for immune dysregulation, polyendocrinopathy, enteropathy, X-linked (IPEX) syndrome. J Allergy Clin Immunol. (2016) 137:953–5.e4. 10.1016/j.jaci.2015.09.030 26559324

[161] Barzaghi F Amaya Hernandez LC Neven B Ricci S Kucuk ZY Bleesing JJ. Long-term follow-up of IPEX syndrome patients after different therapeutic strategies: an international multicenter retrospective study. J Allergy Clin Immunol. (2018) 141:1036–49.e5. 10.1016/j.jaci.2017.10.041 29241729 PMC6050203

[162] Lucas CL Chandra A Nejentsev S Condliffe AM Okkenhaug K. PI3Kδ and primary immunodeficiencies. Nat Rev Immunol. (2016) 16:702–14. 10.1038/nri.2016.93 27616589 PMC5291318

[163] Angulo I Vadas O Garcon F Banham-Hall E Plagnol V Leahy TR. Phosphoinositide 3-kinase gene mutation predisposes to respiratory infection and airway damage. Science. (2013) 342:866–71. 10.1126/science.1243292 24136356 PMC3930011

[164] Michalovich D Nejentsev S. Activated PI3 kinase delta syndrome: from genetics to therapy. Front Immunol. (2018) 9:369. 10.3389/fimmu.2018.00369 29535736 PMC5835040

[165] Coulter TI Chandra A Bacon CM Babar J Curtis J Screaton N. Clinical spectrum and features of activated phosphoinositide 3-kinase δ syndrome: a large patient cohort study. J Allergy Clin Immunol. (2017) 139:597–606.e4. 10.1016/j.jaci.2016.06.021 27555459 PMC5292996

[166] Elkaim E Neven B Bruneau J Mitsui-Sekinaka K Stanislas A Heurtier L. Clinical and immunologic phenotype associated with activated phosphoinositide 3-kinase δ syndrome 2: a cohort study. J Allergy Clin Immunol. (2016) 138:210–18.e9. 10.1016/j.jaci.2016.03.022 27221134

[167] Nademi Z Slatter MA Dvorak CC Neven B Fischer A Suarez F. Hematopoietic stem cell transplant in patients with activated PI3K delta syndrome. J Allergy Clin Immunol. (2017) 139:1046–9. 10.1016/j.jaci.2016.09.040 27847301

[168] Okano T Imai K Tsujita Y Mitsuiki N Yoshida K Kamae C. Hematopoietic stem cell transplantation for progressive combined immunodeficiency and lymphoproliferation in patients with activated phosphatidylinositol-3-OH kinase δ syndrome type 1. J Allergy Clin Immunol. (2019) 143:266–75. 10.1016/j.jaci.2018.04.032 29778502

[169] van de Veerdonk FL Plantinga TS Hoischen A Smeekens SP Joosten LAB Gilissen C. STAT1 mutations in autosomal dominant chronic mucocutaneous candidiasis. N Engl J Med. (2011) 365:54–61. 10.1056/NEJMoa1100102 21714643

[170] Toubiana J Okada S Hiller J Oleastro M Lagos Gomez M Aldave Becerra JC. Heterozygous STAT1 gain-of-function mutations underlie an unexpectedly broad clinical phenotype. Blood. (2016) 127:3154–64. 10.1182/blood-2015-11-679902 27114460 PMC4920021

[171] Zerbe CS Marciano BE Katial RK Santos CB Adamo N Hsu AP. Progressive multifocal leukoencephalopathy in primary immune deficiencies: Stat1 gain of function and review of the literature. Clin Infect Dis. (2016) 62:986–94. 10.1093/cid/civ1220 26743090 PMC4803104

[172] Dadak M Jacobs R Skuljec J Jirmo AC Yildiz Ö Donnerstag F. Gain-of-function STAT1 mutations are associated with intracranial aneurysms. Clin Immunol. (2017) 178:79–85. 10.1016/j.clim.2017.01.012 28161409

[173] Leiding JW Okada S Hagin D Abinun M Shcherbina A Balashov DN. Hematopoietic stem cell transplantation in patients with gain-of-function signal transducer and activator of transcription 1 mutations. J Allergy Clin Immunol. (2018) 141:704–17.e5. 10.1016/j.jaci.2017.03.049 28601685 PMC7802430

[174] Flanagan SE Haapaniemi E Russell MA Caswell R Allen HL De Franco E. Activating germline mutations in STAT3 cause early-onset multi-organ autoimmune disease. Nat Genet. (2014) 46:812–4. 10.1038/ng.3040 25038750 PMC4129488

[175] Milner JD Vogel TP Forbes L Ma CA Stray-Pedersen A Niemela JE. Early-onset lymphoproliferation and autoimmunity caused by germline STAT3 gain-of-function mutations. Blood. (2015) 125:591–9. 10.1182/blood-2014-09-602763 25359994 PMC4304103

[176] Forbes LR Vogel TP Cooper MA Castro-Wagner J Schussler E Weinacht KG Jakinibs for the treatment of immune dysregulation in patients with gain-of-function signal transducer and activator of transcription 1 (STAT1) or STAT3 mutations. J Allergy Clin Immunol. (2018) 142:1665–9. 10.1016/j.jaci.2018.07.020 30092289 PMC6322659

[177] Kuehn HS Ouyang W Lo B Deenick EK Niemela JE Avery DT. Immune dysregulation in human subjects with heterozygous germline mutations in CTLA4. Science. (2014) 345:1623–7. 10.1126/science.1255904 25213377 PMC4371526

[178] Schubert D Bode C Kenefeck R Hou TZ Wing JB Kennedy A. Autosomal dominant immune dysregulation syndrome in humans with CTLA4 mutations. Nat Med. (2014) 20:1410–6. 10.1038/nm.3746 25329329 PMC4668597

[179] Schwab C Gabrysch A Olbrich P Patiño V Warnatz K Wolff D. Phenotype, penetrance, and treatment of 133 cytotoxic T-lymphocyte antigen 4-insufficient subjects. J Allergy Clin Immunol. (2018) 142:1932–46. 10.1016/j.jaci.2018.02.055 29729943 PMC6215742

[180] Slatter MA Engelhardt KR Burroughs LM Arkwright PD Nademi Z Skoda-Smith S. Hematopoietic stem cell transplantation for CTLA4 deficiency. J Allergy Clin Immunol. (2016) 138:615–19.e1. 10.1016/j.jaci.2016.01.045 27102614

[181] Lopez-Herrera G Tampella G Pan-Hammarström Q Herholz P Trujillo-Vargas CM Phadwal K. Deleterious mutations in LRBA are associated with a syndrome of immune deficiency and autoimmunity. Am J Hum Genet. (2012) 90:986–1001. 10.1016/j.ajhg.2012.04.015 22608502 PMC3370280

[182] Bakhtiar S Gámez-Díaz L Jarisch A Soerensen J Grimbacher B Belohradsky B. Treatment of infantile inflammatory bowel disease and autoimmunity by allogeneic stem cell transplantation in LPS-responsive beige-like anchor deficiency. Front Immunol. (2017) 8:52. 10.3389/fimmu.2017.00052 28197149 PMC5281554

[183] Tesi B Priftakis P Lindgren F Chiang SCC Kartalis N Löfstedt A. Successful hematopoietic stem cell transplantation in a patient with LPS-responsive beige-like anchor (LRBA) gene mutation. J Clin Immunol. (2016) 36:480–9. 10.1007/s10875-016-0289-y 27146671

[184] Seidel MG Hirschmugl T Gamez-Diaz L Schwinger W Serwas N Deutschmann A. Long-term remission after allogeneic hematopoietic stem cell transplantation in LPS-responsive beige-like anchor (LRBA) deficiency. J Allergy Clin Immunol. (2015) 135:1384–90.e1-8. 10.1016/j.jaci.2014.10.048 25539626 PMC4429722

[185] Gámez-Díaz L August D Stepensky P Revel-Vilk S Seidel MG Noriko M. The extended phenotype of LPS-responsive beige-like anchor protein (LRBA) deficiency. J Allergy Clin Immunol. (2016) 137:223–30. 10.1016/j.jaci.2015.09.025 26768763

[186] Panchal N Booth C Cannons JL Schwartzberg PL. X-Linked lymphoproliferative disease type 1: a clinical and molecular perspective. Front Immunol. (2018) 9:666. 10.3389/fimmu.2018.00666 29670631 PMC5893764

[187] Gross TG Filipovich AH Conley ME Pracher E Schmiegelow K Verdirame JD. Cure of X-linked lymphoproliferative disease (XLP) with allogeneic hematopoietic stem cell transplantation (HSCT): report from the XLP registry. Bone Marrow Transplant. (1996) 17:741–4. 8733691

[188] Lankester AC Visser LFA Hartwig NG Bredius RGM Gaspar HB van der Burg M. Allogeneic stem cell transplantation in X-linked lymphoproliferative disease: two cases in one family and review of the literature. Bone Marrow Transplant. (2005) 36:99–105. 10.1038/sj.bmt.1705016 15908972

[189] Booth C Gilmour KC Veys P Gennery AR Slatter MA Chapel H. X-linked lymphoproliferative disease due to SAP/SH2D1A deficiency: a multicenter study on the manifestations, management and outcome of the disease. Blood. (2011) 117:53–62. 10.1182/blood-2010-06-284935 20926771 PMC3374620

[190] Marsh RA Bleesing JJ Chandrakasan S Jordan MB Davies SM Filipovich AH. Reduced-intensity conditioning hematopoietic cell transplantation is an effective treatment for patients with SLAM-associated protein deficiency/X-linked lymphoproliferative disease type 1. Biol Blood Marrow Transplant. (2014) 20:1641–5. 10.1016/j.bbmt.2014.06.003 24923536

[191] Speckmann C Lehmberg K Albert MH Damgaard RB Fritsch M Gyrd-Hansen M. X-linked inhibitor of apoptosis (XIAP) deficiency: the spectrum of presenting manifestations beyond hemophagocytic lymphohistiocytosis. Clin Immunol. (2013) 149:133–41. 10.1016/j.clim.2013.07.004 23973892

[192] Marsh RA Rao K Satwani P Lehmberg K Muller I Li D. Allogeneic hematopoietic cell transplantation for XIAP deficiency: an international survey reveals poor outcomes. Blood. (2013) 121:877–83. 10.1182/blood-2012-06-432500 23131490 PMC5162550

[193] Worth AJJ Nikolajeva O Chiesa R Rao K Veys P Amrolia PJ. Successful stem cell transplant with antibody-based conditioning for XIAP deficiency with refractory hemophagocytic lymphohistiocytosis. Blood. (2013) 121:4966–8. 10.1182/blood-2013-01-478735 23766462

[194] Tsuma Y Imamura T Ichise E Sakamoto K Ouchi K Osone S. Successful treatment of idiopathic colitis related to XIAP deficiency with allo-HSCT using reduced-intensity conditioning. Pediatr Transplant. (2015) 19:E25–8. 10.1111/petr.12405 25412586

[195] Chellapandian D Krueger J Schechter T Gassas A Weitzman S Naqvi A. Successful allogeneic hematopoietic stem cell transplantation in XIAP deficiency using reduced-intensity conditioning. Pediatr Blood Cancer. (2016) 63:355–7. 10.1002/pbc.25756 26398727

